# Overlapping forms of granulomatosis with polyangiitis and eosinophilic granulomatosis with polyangiitis: Insights from a European multicenter study

**DOI:** 10.1111/joim.70056

**Published:** 2025-12-09

**Authors:** Federica Pallotti, Camille Mettler, Matthias Papo, Michele Iudici, Roberto Padoan, Boris Sorin, Francesca Regola, Franco Franceschini, Sergey Moiseev, Pavel Novikov, Mario Andrea Piga, Gianluca Moroncini, Silke R. Brix, Abdul Hadi Kafagi, Samuel Deshayes, Achille Aouba, Julien Campagne, Paolo Delvino, Jan Willem Cohen Tervaert, Luisa Brussino, Martin Michaud, Nils Venhoff, Federico Alberici, Claudia Iannone, Sophie Rosenstingl, Marin Moutel, Jean‐Marc Galempoix, Vincent Cottin, Clara Jaccard, Diane Riehl, Paul Legendre, Anne Claire Billet, Paola Parronchi, Luca Quartuccio, Vitor Teixeira, Allyson Egan, David Jayne, Enrico Tombetti, Marco Caminati, Christian Pagnoux, Alexis Regent, Marc Ruivard, Loïc Guillevin, Xavier Puéchal, Benjamin Terrier, the French Vasculitis Study Group (FVSG)

**Affiliations:** ^1^ Department of Internal Medicine Cochin Hospital National Referral Center for Rare Systemic Autoimmune Diseases Paris University Paris France; ^2^ Department of Internal Medicine & Clinical Immunology Caen‐Normandy University Hospital Caen France; ^3^ Department of Internal Medicine Bichat Claude‐Bernard Hospital Paris University Paris France; ^4^ Department of Internal Medicine Division of Rheumatology Geneva University Hospitals Geneva Switzerland; ^5^ Department of Medicine DIMED Rheumatology Unit University of Padua Padua Italy; ^6^ Rheumatology and Clinical Immunology Unit Department of Clinical and Experimental Sciences ASST Spedali Civili and University of Brescia Brescia Italy; ^7^ Tareev Clinic of Internal Disease Sechenov First Moscow State Medical University Moscow Russia; ^8^ Clinica Medica Department of Internal Medicine Marche University Hospital Ancona Italy; ^9^ Department of Clinical and Molecular Sciences Marche Polytechnic University Ancona Italy; ^10^ National PhD Course in Precision Medicine in Rare Diseases University of Palermo Palermo Italy; ^11^ Manchester University NHS Foundation Trust Manchester UK; ^12^ Department of Internal Medicine Robert Schuman Hospital Metz France; ^13^ School of Medicine University of Milano‐Bicocca Milan Italy; ^14^ Rheumatology Unit IRCCS San Gerardo dei Tintori Monza Italy; ^15^ Department of Medicine University of Alberta Edmonton Alberta Canada; ^16^ School for Mental Health and Neuroscience Maastricht University Maastricht the Netherlands; ^17^ Department of Medical Sciences University of Torino & Mauriziano Hospital Torino Italy; ^18^ Department of Internal Medicine Clinique Saint‐Exupery Toulouse France; ^19^ Department of Rheumatology and Clinical Immunology Faculty of Medicine Medical Center—University of Freiburg Freiburg Germany; ^20^ Nephrology Unit University of Brescia ASST Spedali Civili Brescia Italy; ^21^ Department of Rheumatology and Medical Sciences ASST Gaetano Pini‐CTO Milan, and Department of Clinical Sciences and Community Health University of Milan Milan Italy; ^22^ Department of Internal Medicine Compiègne Noyon Hospital Compiègne France; ^23^ Department of Internal Medicine Infectious Diseases and Clinical Immunology Reims University Hospital Reims France; ^24^ Department of Internal Medicine Charleville‐Mézières Hospital Charleville‐Mézières France; ^25^ National Coordinating Reference Centre for Rare Lung Diseases Department of Pneumology Louis‐Pradel Hospital Hospices Civils de Lyon France; ^26^ Department of Internal Medicine Gaston‐Bourret Territorial Hospital Nouméa New Caledonia; ^27^ Department of Internal Medicine La Seyne sur Mer Hospital La Seyne sur Mer France; ^28^ Department of Internal Medicine Centre Hospitalier Le Mans Le Mans France; ^29^ Department of Internal Medicine Hôpital Edouard Herriot Hospices Civils de Lyon Lyon France; ^30^ Department of Experimental and Clinical Medicine University of Florence Florence Italy; ^31^ Rheumatology Division Department of Medicine (DMED) University of Udine Udine Italy; ^32^ Department of Rheumatology Universidade Local de Saúde do Algarve Faro Portugal; ^33^ Trinity Health Kidney Centre Tallaght University Hospital Dublin Ireland; ^34^ Department of Medicine University of Cambridge Cambridge UK; ^35^ Department of Internal Medicine ASST Fatebenefratelli‐Sacco Fatebenefratelli Hospital Milan Italy; ^36^ Allergy Unit and Asthma Center Department of Medicine Integrated University Hospital and University of Verona Verona Italy; ^37^ Mount Sinai Hospital Toronto Ontario Canada; ^38^ Department of Internal Medicine Clermont‐Ferrand University Hospital Clermont‐Ferrand France

**Keywords:** ANCA‐associated vasculitides, eosinophilic granulomatosis with polyangiitis, granulomatosis with polyangiitis, overlapping forms

## Abstract

**Background:**

Granulomatosis with polyangiitis (GPA) and eosinophilic granulomatosis with polyangiitis (EGPA) are distinct forms of antineutrophil cytoplasm antibody (ANCA)‐associated vasculitis (AAV). Increasing evidence suggests overlapping features, particularly in proteinase 3 (PR3)‐ANCA‐positive EGPA and GPA with eosinophilia. This study aimed to characterize overlapping EGPA/GPA forms and assess their clinical and therapeutic implications.

**Methods:**

We conducted a European, multicenter, observational study, including 135 patients with overlapping EGPA/GPA features. Definitions were based on ACR/EULAR classification criteria and other clinical and biological findings. Clinical, biological, and histological characteristics were analyzed using unsupervised hierarchical clustering approach. Comparisons were made with established EGPA and GPA control cohorts.

**Results:**

Three clusters emerged: Cluster 1, a hybrid phenotype (pulmonary nodules, PR3‐ANCA positivity, high relapse rate); Cluster 2, a systemic inflammatory phenotype (constitutional symptoms, PR3‐ANCA positivity, moderate renal involvement); and Cluster 3, a severe vasculitis form (severe renal disease, alveolar hemorrhage). Including typical EGPA and GPA control cohorts revealed two main clusters a posteriori: an EGPA cluster and a GPA cluster. Cluster 1 overlapped with both EGPA and GPA clusters, whereas Clusters 2 and 3 predominantly aligned with GPA. Kaplan–Meier analysis revealed that Cluster 1 and the typical EGPA cohort had the best overall survival, whereas Cluster 3 had the poorest survival. Relapse‐free survival was highest in typical EGPA and poorest in Cluster 3 and typical GPA.

**Conclusion:**

This study delineates the heterogeneity of EGPA/GPA overlap and underscores the need for personalized treatment approaches. Future prospective studies should explore targeted therapies, including rituximab and IL‐5 blockade, in these overlapping AAV subtypes.

## Graphical abstract

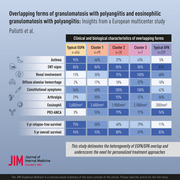

**Abbreviations**
AAV: ANCA‐associated vasculitis
ACR: American College of Rheumatology
ANCA: anti‐neutrophil cytoplasm antibody(ies)
AZA: azathioprine
BVAS: Birmingham Vasculitis Activity Score
CHCC: Chapel Hill Consensus Conference
CYC: cyclophosphamide
DAH: diffuse alveolar hemorrhage
EGPA: eosinophilic granulomatosis with polyangiitis
EMEA: European Medicines Agency
ENT: ear nose throat
EULAR: European Alliance of Associations for Rheumatology
FFS: five factor score
FVSG: French Vasculitis Study Group
GCs: glucocorticoids
GWAS: Genome Wide Association Study
HLA: human leukocyte antigens
IL: interleukin
IQR: interquartile range
MPA: microscopic polyangiitis
MPO: myeloperoxidase
MTX: methotrexate
OS: overall survival
PLEX: plasma exchange
PR3: proteinase 3
RTX: rituximab
RFS: relapse‐free survival

## Introduction

Antineutrophil cytoplasm antibody (ANCA)‐associated vasculitis (AAV) are necrotizing small‐vessel vasculitides characterized by ANCA targeting proteinase 3 (PR3) or myeloperoxidase (MPO) [1]. The 2012 revised Chapel Hill Consensus Conference classified AAV into granulomatosis with polyangiitis (GPA), microscopic polyangiitis (MPA), and eosinophilic granulomatosis with polyangiitis (EGPA) [1]. ANCAs are detected in approximately 90% of GPA cases, predominantly PR3‐ANCA, and in 60%–80% of MPA cases, mostly MPO‐ANCA [2, 3]. In contrast, only 30%–40% of EGPA patients are ANCA‐positive, primarily MPO‐ANCA, with PR3‐ANCA being rare (1%–3%) [3, 4, 5]. Clinically, PR3‐ANCA‐positive AAVs are typically associated with acute renal and granulomatous lesions of the respiratory tract, as well as a higher relapse rate, whereas MPO‐ANCA‐positive AAVs more often present with indolent fibrotic renal and interstitial lung involvement [6, 7]. EGPAs, however, exhibit distinct features, including eosinophilia, late‐onset asthma, and nasal polyposis, with two main phenotypes: MPO‐ANCA‐positive EGPA, dominated by vasculitic lesions, and ANCA‐negative EGPA, characterized by eosinophilic manifestations [8, 9, 10, 11]. Renal involvement, which is common in GPA and MPA, is observed in only 5%–25% of EGPA cases, predominantly in those with MPO‐ANCA positivity [12, 13, 14].

Although these classifications provide useful diagnostic frameworks, overlap between GPA and EGPA has become increasingly recognized. PR3‐ANCA‐positive EGPAs have been reported to share features with GPA, including higher relapse rates, whereas GPAs with eosinophilia tend to exhibit increased disease activity and more frequent eosinophil‐mediated damages [4, 15, 16]. These overlapping forms raise critical questions regarding classification and management, as therapeutic approaches differ significantly. Rituximab is considered the gold standard for GPA [15, 17, 18, 19, 20, 21, 22, 23] but supporting evidence in EGPA remains limited [23, 24]. Conversely, anti‐IL‐5/IL‐5R therapies are approved for EGPA but remain largely unexplored in GPA with eosinophilia [25, 26, 27]. Similarly, the efficacy of avacopan, demonstrated in GPA and MPA [23, 28], has yet to be evaluated in EGPA.

This study aims to clarify the heterogeneity of EGPA/GPA overlap by describing their clinical and biological characteristics, disease severity, and therapeutic management, and by comparing these features with typical EGPA and GPA cases.

## Materials and methods

### Study design

We conducted a European, observational, retrospective, multicenter study to investigate overlapping forms of EGPA and GPA. Data were obtained from 46 tertiary care centers across eight countries (France, Italy, the United Kingdom, Russia, Germany, Belgium, the Netherlands, and Portugal). This study was conducted in accordance with the Good Clinical Practice protocol and the principles of the Declaration of Helsinki and received approval from the local Institutional Review Board.

### Patients

We included patients with overlapping features of EGPA and GPA at diagnosis, based on multiple definitions and clinical situations, as there are currently no standardized criteria for this subgroup. Inclusion criteria required comprehensive clinical data, eosinophil count, and ANCA status. The following definitions were applied (Table S1): (1) patients fulfilling both 2022 ACR/EULAR classification criteria for GPA [23, 29] and EGPA [23, 30]; (2) patients meeting the 2022 ACR/EULAR classification criteria for EGPA but also presenting PR3‐ANCA and/or surrogate GPA markers (pulmonary nodules or cavitations >1 month, bronchial stenosis, bloody nasal discharge and crusting >1 month, or nasal ulceration, otitis media or mastoiditis >3 months, retro‐orbital mass or inflammation, subglottic stenosis, saddle nose deformity or destructive sinonasal disease) as proposed in the European Medicines Agency (EMA) algorithm [31]; (3) patients meeting the 2022 ACR/EULAR criteria for GPA or the EMA diagnostic algorithm, with eosinophils >1000/mm^3^ at diagnosis; (4) patients with a clinician‐diagnosed AAV who did not meet the 2022 ACR/EULAR criteria for EGPA or GPA but who presented with eosinophils >1000/mm^3^ and PR3‐ANCA and/or surrogate GPA markers.

Standardized case report forms captured demographic, clinical, laboratory, radiologic, and histologic data. Laboratory variables included serum creatinine, eosinophil count, and ANCA status. Disease activity was assessed using the Birmingham Vasculitis Activity Score (BVAS) 2003 [32]. The 1996 Five Factor Score (FFS) was also recorded at baseline [33].

### Outcomes

As recommended in the 2022 updated EULAR guidelines [23], relapses were defined as the recurrence of disease following remission, regardless of ongoing immunosuppressive therapy, with clinical manifestations attributable to active vasculitis (BVAS >0) and the need of treatment escalation [34]. Specifically, a major relapse was defined as the recurrence of disease activity requiring an increase in prednisone‐equivalent dose ≥20 mg/day and/or initiation or modification of immunosuppressive therapy, whereas minor relapse was defined as the recurrence of disease activity requiring an increase in prednisone dose <20 mg/day without any change in immunosuppressants [20, 23, 35]. Clinical and biological findings were recorded for each relapse. Isolated glucocorticoid‐dependent asthma and/or ENT manifestations were defined as symptoms requiring a prednisone dose >7.5 mg/day without any other concomitant vasculitis symptoms. Remission was defined as the absence of disease activity with a BVAS of 0.

### EGPA and GPA control cohorts

To compare overlapping forms with typical EGPA and GPA cases, control patients were selected from previously published FVSG studies: Typical EGPA patients were identified from the study by Papo et al. [4], and typical GPA patients from the FVSG registry analyzed by Iudici et al. [36]. Only cases with available ANCA status and blood eosinophil count were included. The EGPA control cohort included patients fulfilling the 2022 ACR/EULAR classification criteria for EGPA (score ≥6) [30] and excluded patients with anti‐PR3 antibodies and pulmonary nodules. The GPA control cohort included patients fulfilling the 2022 ACR/EULAR classification criteria for GPA (score ≥5) [29] and excluded patients with eosinophilia >1000/mm^3^ (Fig. S1).

### Statistical analysis

Descriptive analyses were performed, with categorical variables reported as counts and percentages, and continuous variables summarized as medians with interquartile ranges (IQR). To identify clusters of patients with overlapping forms of EGPA and GPA, we used unsupervised multiple correspondence analysis. We selected the following variables to capture vasculitis manifestations at diagnosis: age, sex, systemic signs, pulmonary signs as asthma, pulmonary nodules or cavitary lesions, pulmonary consolidation, alveolar hemorrhage, ENT manifestations as subglottic stenosis, nasal polyps, nasal crusting, sinusitis, otologic signs, chondritis, scleritis/episcleritis, cutaneous manifestations as purpura, urticaria, livedo, subcutaneous nodules, renal involvement, myopericarditis, mononeuritis multiplex, central nervous system involvement, gastrointestinal involvement, arthralgia, PR3‐ANCA, MPO‐ANCA, C‐reactive protein (CRP), serum creatinine, and eosinophil count.

Hierarchical clustering on principal components was conducted using the FactoMineR package in R and RStudio to generate homogeneous patient clusters [37]. The optimal number of clusters was determined visually from the dendrogram and based on inertia gain. Subgroup comparisons were carried out using the Kruskal–Wallis rank sum test for continuous variables and Pearson's *χ* ^2^ test for categorical variables if expected cell counts exceeded 5; otherwise, Fisher's exact test was applied. To describe the outcomes within the identified clusters, we calculated overall survival (OS) and relapse‐free survival (RFS) proportion at 1, 3, and 5 years of follow‐up. Kaplan–Meier survival curves were plotted for each cluster. A Fine‐Gray model (cmprsk package) was used in sensitivity analysis to assess the cumulative risk of relapse in the presence of competing events such as death.

Finally, a second clustering analysis was performed, incorporating the EGPA and GPA control cohorts. This analysis included only variables available in both datasets, excluding ENT stenosis, ENT polyps, chondritis, and scleritis/episcleritis, which were not documented in the control cohorts. The distributions of patients according to the inclusion criteria definition and the resulting clusters, along with their respective survival curves, were analyzed.

## Results

### Patient characteristics

**Fig. 1:**
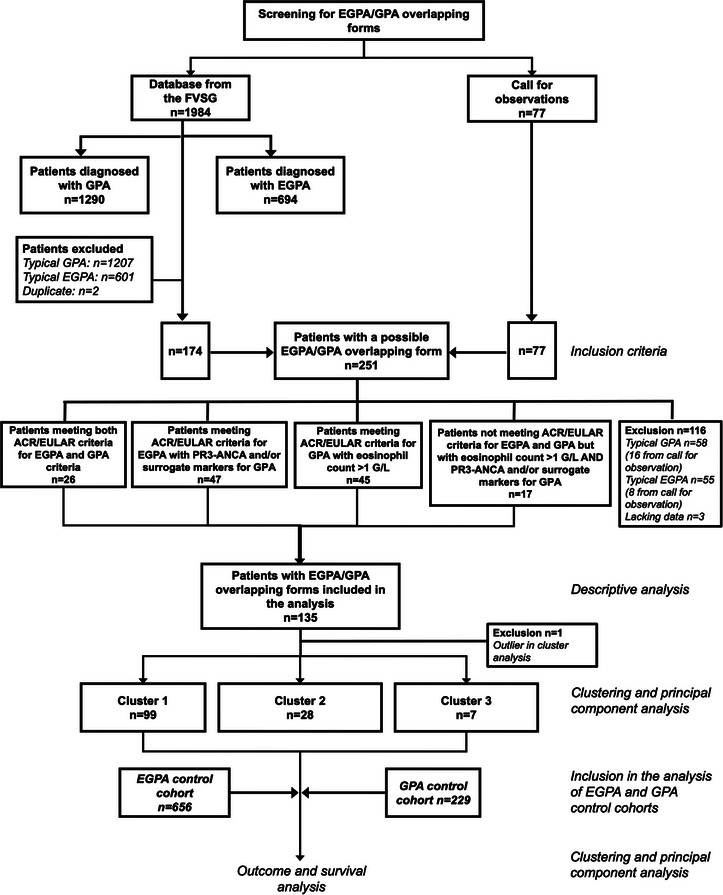
**Flow chart of the study**. ACR, American College of Rheumatology; EGPA, eosinophilic granulomatosis with polyangiitis; EULAR, European Alliance of Associations for Rheumatology; FVSG, French Vasculitis Study Group; GPA, granulomatosis with polyangiitis.

We analyzed 174 patients from the FVSG cohort and 77 from the European call for observations to assess inclusion and exclusion criteria. Out of 251 patients screened, 135 fulfilled at least one inclusion criterion for overlapping EGPA and GPA features and were included in the analysis (Fig. 1). Patients were diagnosed between 1985 and 2024. Baseline characteristics are summarized in Table 1 and detailed by inclusion definition in Table S2. The mean age at diagnosis was 52.5 years (IQR 41.2–64), with 51 female patients (37.8%). Pulmonary manifestations were the most frequent (90.4%), including asthma and nodules (both 56.3%), followed by ENT involvement (84.4%), predominantly chronic sinusitis (61.5%). Renal and cardiac involvement were observed in 47.4% and 23% of cases, respectively. ANCA positivity was detected in 104 patients (77%), including PR3‐ANCA in 82 (60.7%) and MPO‐ANCA in 22 (16.3%). The median eosinophil count was 2400/mm^3^ (IQR 1400–6000/mm^3^). According to our inclusion definitions, the 2022 ACR/EULAR classification criteria were met by all the patients for the first (*n* = 26) and the second (*n* = 47) definitions, by 88.9% of those in the third definition (40/45 patients, five patients of whom fulfilled the EMA algorithm), and by none of the 17 patients in the fourth definition (Table S2).

**Table 1:** Main clinical and biological characteristics of the patients with EGPA‐GPA overlapping forms included in the study.

	Overall (*n* = 135)
Female	51 (37.8%)
Age at diagnosis (years)	52.5 [41.2–64]
*ANCA status*	104 (77%)
PR3‐ANCA	82 (60.7%)
MPO‐ANCA	22 (16.3%)
*Clinical manifestations*	
Constitutional symptoms	95 (70.4%)
Arthralgia	85 (63%)
Myalgia	55 (40.7%)
Lung involvement	122 (90.4%)
Asthma	76 (56.3%)
Nodule	76 (56.3%)
Consolidation	57 (42.2%)
Cavitation	10 (7.4%)
Alveolar hemorrhage	27 (20%)
Subglottic‐bronchial stenosis	9 (6.7%)
ENT involvement	114 (84.4%)
Chronic sinusitis	83 (61.5%)
Nasal crusts	71 (52.5%)
Nasal polyps	33 (24.4%)
Otologic	22 (16.3%)
Chondritis	8 (5.9%)
Cutaneous involvement	70 (51.9%)
Purpura	38 (28.1%)
Urticaria	15 (11.1%)
Nodules	9 (6.7%)
Livedo	7 (5.2%)
Renal involvement	64 (47.4%)
Peripheral neuropathy	54 (40%)
CNS involvement	11 (8.1%)
Cardiovascular involvement	31 (23%)
Myocarditis	13 (9.6%)
Pericarditis	12 (8.9%)
Ocular involvement	24 (17.8%)
Gastrointestinal involvement	23 (17%)
FFS score ≥1	30 (22.2%)
BVAS	23 [17–28.5]
*Biological features*	
CRP (mg/L)	61 [29–112.5]
Serum creatinine (µmol/L)	88 [72–115]
Eosinophil count (cells/mm^3^)	2400 [1400–6000]
*2022 ACR/EULAR classification criteria*	
EGPA criteria ≥6, *n*	72 (53.3%)
GPA criteria ≥5, *n*	66 (48.9%)

*Note*: Data are presented as median [IQR] or number (proportion).

Abbreviations: ANCA, antineutrophil cytoplasm antibodies; CNS, central nervous system; CRP, C‐reactive protein; ENT, ear–nose–throat; EGPA, eosinophilic granulomatosis with polyangiitis; FFS, Five Factor Score; GPA, granulomatosis with polyangiitis; MPO‐ANCA, anti‐myeloperoxidase antibodies; PR3‐ANCA, anti‐proteinase‐3 antibodies.

### Therapeutic management and outcome

Treatment regimens and outcomes are summarized in Table S3. Remission was achieved in 126 patients (93.3%) following induction therapy, which included glucocorticoids (99.3%), cyclophosphamide (42.2%), and rituximab (17%). Three patients (2.2%) received anti‐IL‐5 as induction therapy. Maintenance therapy included glucocorticoids (57%), azathioprine (29.6%), rituximab (14.8%), methotrexate (14.1%), and anti‐IL‐5/IL‐5R therapies (4.4%).

During follow‐up (median duration 77.9 months, IQR 40.5–121.2), 73 patients (54.1%) experienced relapses: 48 (35.6%) major and 12 (8.9%) minor relapses. Isolated glucocorticoid‐dependent asthma and/or ENT exacerbations occurred in 13 patients (9.6%). RFS was 82% at 1 year, 60.2% at 3 years, and 47.7% at 5 years (Fig. S2). OS was 89.8% at 5 years, with 18 deaths (13.3%), mainly due to severe infections (27.8%) and AAV‐related complications (16.7%). Additional treatments for disease activity were required in 91 patients (67.4%) during follow‐up, including rituximab in 27 patients (20%) and anti‐IL‐5/IL‐5R therapy in 14 patients (10.4%; mepolizumab in 11 and benralizumab in 3) (Table S3).

### Clustering analysis of EGPA/GPA overlapping forms

**Fig. 2:**
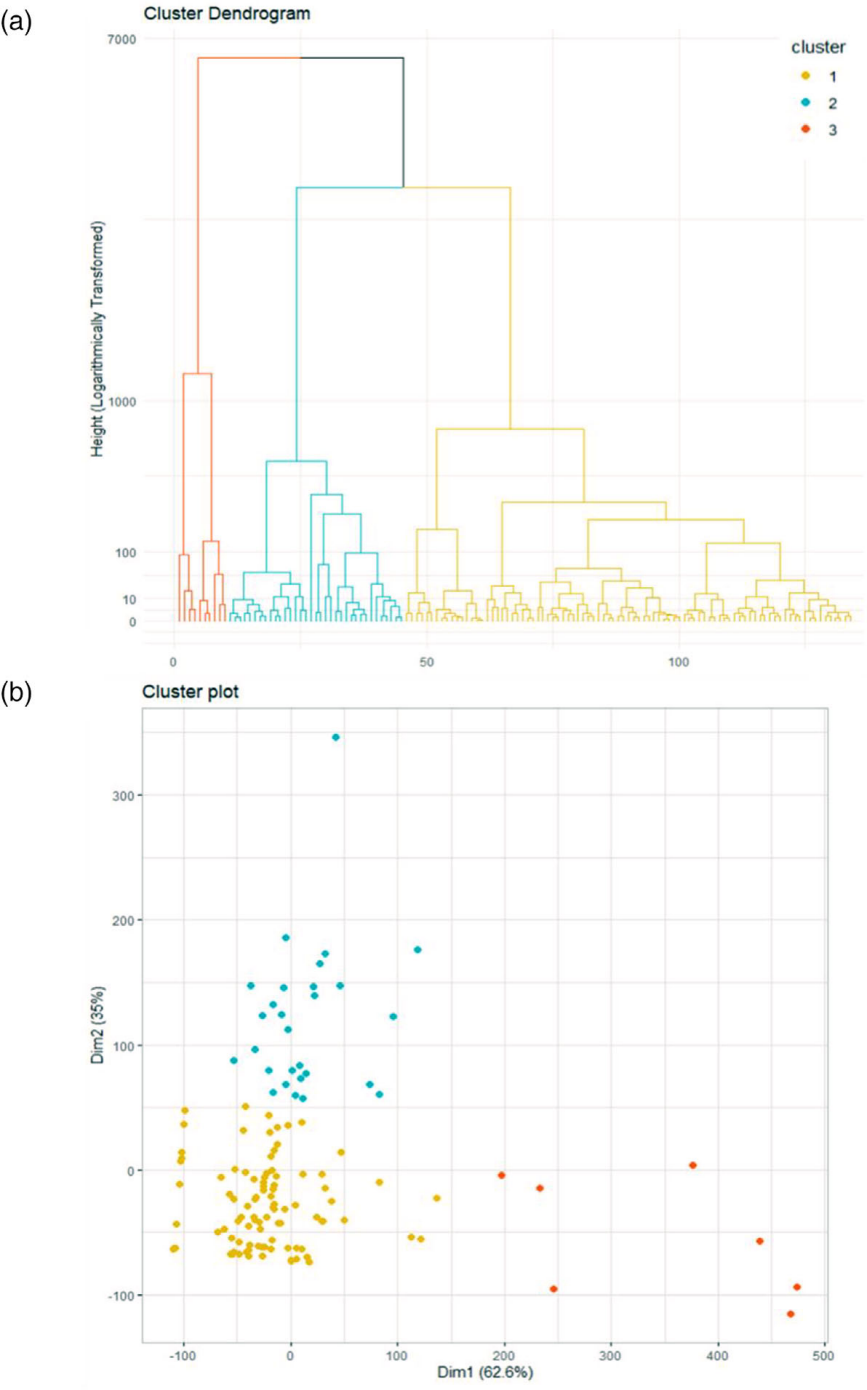
**Hierarchical cluster analysis with dendrogram (a) and principal component analysis (b) of patients with** eosinophilic granulomatosis with polyangiitis **(EGPA)/**granulomatosis with polyangiitis **(GPA) overlapping forms**. Panel a. Dendrogram showing sample clustering based on hierarchical clustering with logarithmic transformation of the distance metric. Three major clusters were identified and are highlighted in distinct colors. Panel b. Principal component projection of the clustered samples, illustrating the spatial separation of the three groups along the first two dimensions (Dim1: 62.6% variance explained, Dim2: 35%).

Unsupervised hierarchical clustering identified three distinct patient clusters (Table 2, Fig. 2). One patient was excluded as an outlier. The distribution of patients from the initial inclusion definitions into cluster is shown in Table S4, and histological findings are available in Table S5.

**Table 2:** Clinical and biological characteristics of the three clusters of patients.

	Cluster 1 *(n* = 99)	Cluster 2 *(n* = 28)	Cluster 3 *(n* = 7)	*p* value
**Gender (female)**	42 (42.4%)	8 (28.6%)	1 (14.3%)	0.219
**Age (years)**	50 [39.5–64]	55 [48–64.5]	44 [35–55.5]	0.296
***Vasculitis manifestations***				
**Constitutional symptoms**	**59 (59.6%)**	**28 (100%)**	**7 (100%)**	**<0.001**
**Pulmonary manifestations**	**94 (94.9%)**	**21 (75%)**	**6 (85.7%)**	**0.008**
Nodule/cavitation	61 (61.6%)	12 (42.9%)	3 (42.9%)	0.126
Asthma	**65 (65.7%)**	**8 (28.6%)**	**3 (42.9%)**	**<0.001**
Diffuse alveolar hemorrhage	**17 (17.2%)**	**5 (17.9%)**	**4 (57.1%)**	**0.046**
Condensation	44 (44.4%)	8 (28.6%)	4 (57.1%)	0.192
**ENT manifestations**	83 (83.8%)	24 (85.7%)	6 (85.7%)	1
Sinusitis	64 (64.6%)	15 (53.6%)	4 (57.1%)	0.554
Polyps	27 (27.3%)	5 (17.9%)	1 (14.3%)	0.575
Nasal crusts	43 (43.4%)	17 (60.7%)	2 (28.6%)	0.165
Otological	15 (15.2%)	6 (21.4%)	1 (14.3%)	0.826
Stenosis	3 (3%)	1 (3.6%)	0 (0%)	1
**Chondritis**	6 (6.1%)	2 (7.1%)	0 (0%)	1
**Scleritis/Episcleritis**	8 (8.1%)	4 (14.3%)	1 (14.3%)	0.361
**Arthralgia**	**54 (54.5%)**	**26 (92.9%)**	**4 (57.1%)**	**<0.001**
**Cutaneous manifestations**	51 (51.5%)	13 (46.4%)	5 (71.4%)	0.6
Purpura	24 (24.2%)	11 (39.3%)	2 (28.6%)	0.314
Urticaria	15 (15.2%)	0 (0%)	0 (0%)	0.064
Subcutaneous nodule	**7 (7.1%)**	**0 (0%)**	**2 (28.6%)**	**0.036**
Livedo	5 (5.1%)	2 (7.1%)	0 (0%)	0.763
**Renal involvement**	**35 (35.4%)**	**21 (75%)**	**7 (100%)**	**<0.001**
**Myopericarditis**	18 (18.2%)	2 (7.1%)	2 (28.6%)	0.221
**Multiple mononeuropathy**	26 (26.3%)	12 (42.9%)	1 (14.3%)	0.146
**CNS involvement**	9 (9.1%)	1 (3.6%)	1 (14.3%)	0.407
**Gastrointestinal**	16 (16.2%)	4 (14.3%)	2 (28.6%)	0.583
***Laboratory features***				
PR3‐ANCA	54 (54.5%)	22 (78.6%)	5 (71.4%)	0.053
MPO‐ANCA	16 (16.2%)	5 (17.9%)	1 (14.3%)	0.913
CRP (mg/L)	**34 [8.5–63]**	**188.5 [149.2–217.8]**	**56 [11–82.5]**	**<0.001**
Creatinine (µmol/L)	**79 [55.5–97.5]**	**102 [80.8–113.5]**	**477 [346.5–562]**	**<0.001**
Eosinophil (cells/mm^3^)	2400 [1400–7000]	1900 [1400–4000]	2100 [1700–5700]	0.574
**Induction treatment**				
Glucocorticoids	94 (94.9%)	24 (85.7%)	7 (100%)	
Methylprednisolone pulse	45 (45.5%)	16 (57.1%)	4 (57.1%)	
Cyclophosphamide	32 (32.3%)	17 (60.7%)	7 (100%)	
Rituximab	18 (18.2%)	5 (17.9%)	0 (0%)	
Plasma exchange	5 (5.1%)	4 (14.3%)	2 (28.6%)	
Azathioprine	7 (7.1%)	0 (0%)	0 (0%)	
Methotrexate	7 (7.1%)	1 (3.6%)	0 (0%)	
Mepolizumab	2 (2%)	1 (3.6%)	0 (0%)	
**Maintenance treatment**				
Rituximab	13 (13.1%)	6 (21.4%)	1 (14.3%)	
Azathioprine	31 (31.3%)	6 (21.4%)	2 (28.6%)	
Methotrexate	13 (13.1%)	6 (21.4%)	0 (0%)	
Mepolizumab	5 (5.1%)	1 (3.6%)	0 (0%)	
Glucocorticoids oral dose, median (mg/day)	5 [0–5]	0 [0–5]	5 [1.5–5]	

*Note*: Data are presented as median [IQR] or number (proportion). Statistically significant *p* values are presented in bold.

Abbreviations: ANCA, antineutrophil cytoplasm antibodies; CNS, central nervous system; CRP, C‐reactive protein; ENT, ear–nose–throat; EGPA, eosinophilic granulomatosis with polyangiitis; FFS, Five Factor Score; GPA, granulomatosis with polyangiitis; MPO‐ANCA, anti‐myeloperoxidase antibodies; PR3‐ANCA; anti‐proteinase‐3 antibodies.

Cluster 1 (*n* = 99 patients, 73.9%) comprised patients with mild‐to‐moderate disease predominantly characterized by pulmonary manifestations (94.9%), including asthma (65.7%) and pulmonary nodules/cavitations (61.6%). ENT involvement was common (83.8%), with nasal crusts (43.4%) and sinusitis (64.6%). Renal involvement was infrequent (35.4%): primarily associated with PR3‐ANCA (71.4%), with 81.3% showing pauci‐immune necrotizing glomerulonephritis. The median eosinophilic count was 2400/mm^3^ (IQR 1400–7000).

Cluster 2 (*n* = 28 patients, 20.9%) presented systemic disease, marked by constitutional symptoms (100%), elevated CRP (median 188.5 mg/L, IQR 149.2–217.8), frequent arthralgia (92.9%), nasal crusts (60.7%), and multiple mononeuropathy (42.9%). Pauci‐immune necrotizing glomerulonephritis was more common (75%) but less severe than in Cluster 3, and PR3‐ANCA was predominant (78.6%).

Cluster 3 (*n* = 7, 5.2%) had the most severe disease with constitutional symptoms (100%), severe renal involvement (100% of pauci‐immune necrotizing glomerulonephritis, median serum creatinine 477 µmol/L, IQR 346.5–562), and frequent pulmonary manifestations (85%), including diffuse alveolar hemorrhage in 57.1% of patients.

Induction therapy was based on intravenous cyclophosphamide in all patients in Cluster 3, in 60.7% of Cluster 2, and in 32.3% of Cluster 1 (Table 2). Rituximab was used in about one‐fifth of patients in Clusters 1 and 2, whereas mepolizumab was rarely administered in Cluster 1. As rituximab became available for induction therapy of AAV only after 2010, we performed an additional analysis restricted to patients diagnosed after 2010, which did not reveal significant differences in treatment patterns (Table S6).

Relapse rates were comparable across clusters (Table 3, Fig. S3). Clinical and biological findings during relapses are reported in Table S7. At relapse, the median eosinophil count was 0/mm^3^ [IQR (0–1000) for Cluster 1, (0–0) for Cluster 2, and (0–600) for Cluster 3]. Although no significant differences were observed, RFS at 1 year was higher in Cluster 2 (88%) and Cluster 1 (83.2%) compared to Cluster 3 (57.1%) (Table 3). This trend persisted at 3 and 5 years. Mortality was significantly higher in Cluster 3 (42.9%) than Cluster 2 (17.9%) and Cluster 1 (9.2%) (*p* = 0.036). The Fine‐Gray model assessing cumulative risk of relapse with death as a competitive event yielded similar results (Fig. S3B).

**Table 3:** Outcomes in terms of relapses, deaths, and overall survival according to cluster groups.

	Cluster 1 (*n* = 99)	Cluster 2 (*n* = 28)	Cluster 3 (*n* = 7)	*p value*
**French vasculitis study group relapse score (FRS)**	3 [2–3]	3 [2–3]	2 [1.5–2]	
**Relapses**	57 (57.6%)	13 (46.4%)	3 (42.9%)	0.46
**Time from induction therapy (months)**	25.4 [11.1–51.7]	26.4 [18.3–37.5]	24.4 [15.2–27.9]	
**Major vasculitis relapse**	35 (35.4%)	10 (35.7%)	3 (42.9%)	
**Minor vasculitis relapse**	11 (11.1%)	1 (3.6%)	0 (0%)	
**Isolated asthma/ENT signs**	11 (11.1%)	2 (7.1%)	0 (0%)	
**Relapse‐free survival**				
**1 year *(n)***	78 (83%)	22 (88%)	4 (57.1%)	0.162
**3 years *(n)***	59 (62.8%)	16 (64%)	2 (28.6%)	0.221
**5 years *(n)***	47 (50%)	12 (48%)	2 (28.6%)	0.587
**Death**	9 (9.2%)	5 (17.9%)	3 (42.9%)	**0.036**
**Overall survival**				
**1 year *(n)***	93 (98.9%)	25 (100%)	5 (71.4%)	**0.008**
**3 years *(n)***	88 (93.6%)	24 (96%)	4 (57.1%)	**0.016**
**5 years *(n)***	87 (92.6%)	22 (88%)	4 (57.1%)	**0.017**

*Note*: Data are presented as median [IQR] or number (proportion). Statistically significant *p* values are presented in bold.

Abbreviation: ENT, ear–nose–throat.

Data on end‐stage renal disease (ESRD) were incomplete in this cohort: serum creatinine at last follow‐up was unavailable for 69 patients, one patient had an eGFR <15 mL/min/1.73 m^2^, and information on dialysis or transplantation was lacking, precluding a reliable evaluation of ESRD as an outcome.

### Comparison with EGPA and GPA control cohorts

**Fig. 3:**
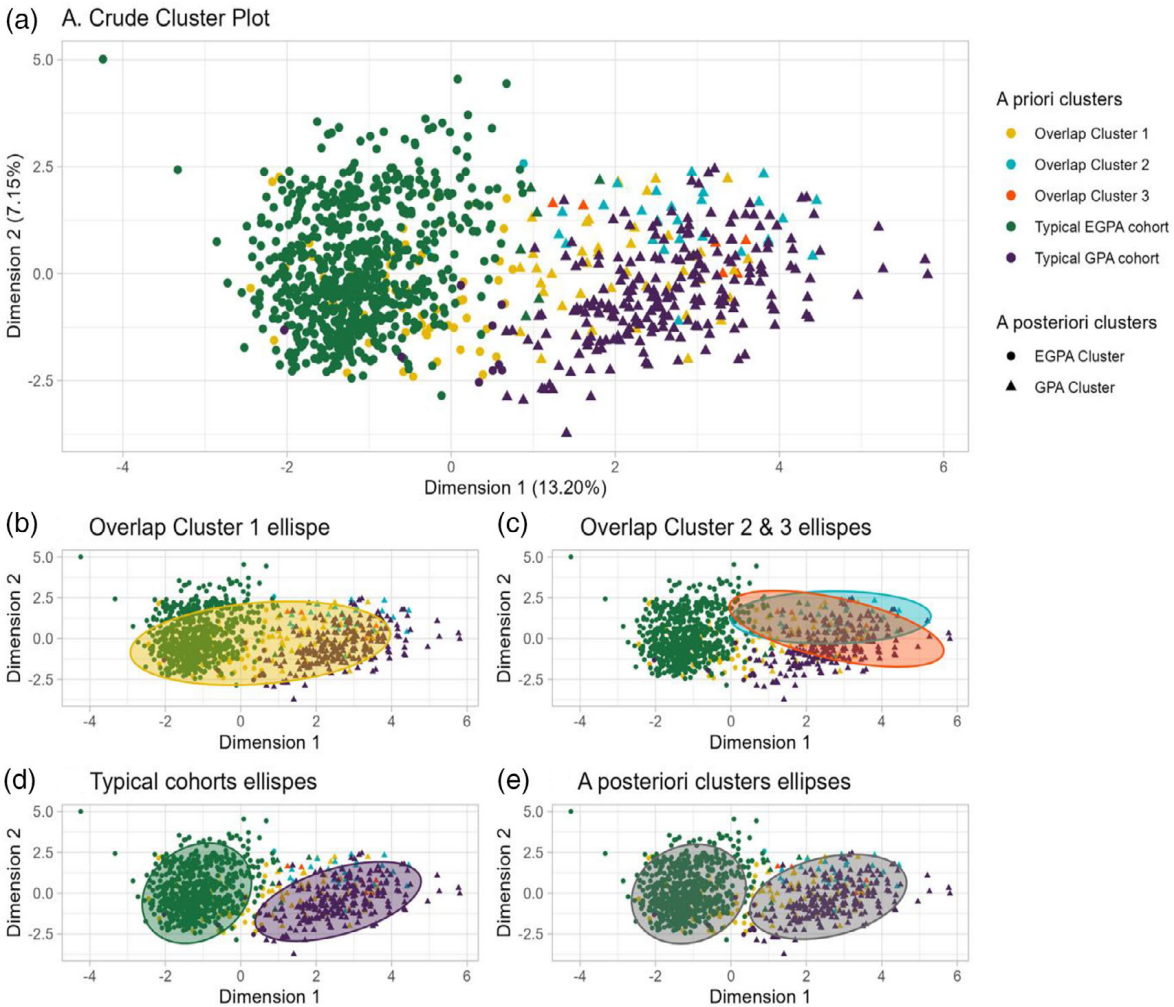
**Hierarchical cluster analysis in principal component between overlapping forms of** eosinophilic granulomatosis with polyangiitis **(EGPA)/**granulomatosis with polyangiitis **(GPA), GPA and EGPA control cohorts. Panel a** displays the distribution of patients according to cluster analysis. Patients with overlap forms of EGPA and GPA are represented by three distinct clusters (Clusters 1–3, shown in different colors). Typical EGPA and GPA control cohorts are also represented by specific colors. In addition, the a posteriori clustering distinguishes two main groups: the EGPA cluster and the GPA cluster, depicted by circles and triangles, respectively. **Panel b** shows Cluster 1 overlapping equally with both of the two main patients’ populations, that is, the a posteriori EGPA (51.1%) and GPA (48.9%) clusters. **Panel c** shows Clusters 2 (89.3%) and 3 (100%) corresponding predominantly to the a posteriori GPA cluster. **Panel d** shows that almost all typical EGPA patients (99.1%) overlapped with the a posteriori EGPA cluster, whereas almost all typical GPA patients (96.1%) overlapped with the a posteriori GPA cluster. **Panel e** illustrates the two a posteriori clusters, that is, the “EGPA cluster” and the “GPA cluster.”

Clusters were compared with typical EGPA (*n* = 656) and GPA (*n* = 229) cohorts (Fig. S1 and Table S8). The mean vectors of variables influencing cluster assignment are shown in Fig. S4. The overall cluster analysis identified two principal groups retrospectively: an “EGPA cluster” comprising almost all typical EGPA patients (99.1%) and a “GPA cluster” comprising almost all typical GPA patients (96.1%) (Fig. 3, Table 4). Cluster 1 overlapped equally with both EGPA (51.1%) and GPA (48.9%) clusters, whereas Clusters 2 (89.3%) and 3 (100%) corresponded predominantly to GPA profiles. Based on the inclusion criteria, patients fulfilling the first definition overlapped equally with both EGPA and GPA clusters (50% vs. 50%) (Table 4). Patients meeting the third and fourth definitions predominantly corresponded to the GPA cluster (88.9% and 87.5%, respectively), whereas most patients included under the second definition corresponded to the EGPA cluster (87.2%).

**Table 4:** Overall cluster analysis between typical EGPA and GPA cohorts, the three clusters, and the four initial definitions.

A posteriori cluster	EGPA cluster	GPA cluster
**Typical cohorts**	*n* = 720	*n* = 299
EGPA (*n* = 656)	650 (99.1%)	6 (0.9%)
GPA (*n* = 229)	9 (3.9%)	220 (96.1%)
**Cluster proportion**		
Cluster 1 (*n* = 99)	51 (51.1%)	48 (48.9%)
Cluster 2 (*n* = 28)	3 (10.7%)	25 (89.3%)
Cluster 3 (*n* = 7)	0 (0%)	7 (100%)
**Initial definition of inclusion criteria**		
Definition 1 (*n* = 26)	13 (50%)	13 (50%)
Definition 2 (*n* = 47)	41 (87.2%)	6 (12.8%)
Definition 3 (*n* = 45)	5 (11.1%)	40 (88.9%)
Definition 4 (*n* = 16)	2 (12.5%)	14 (87.5%)

*Note*: Definition 1 = both GPA and EGPA criteria; Definition 2 = EGPA criteria and PR3‐ANCA ± lung nodules; Definition 3 = GPA criteria and eosinophilia >1000/mm^3^; Definition 4 = AAV diagnosis with PR3‐ANCA and eosinophilia >1000/mm^3^.

Abbreviations: EGPA, eosinophilic granulomatosis with polyangiitis; GPA, granulomatosis with polyangiitis.

**Fig. 4:**
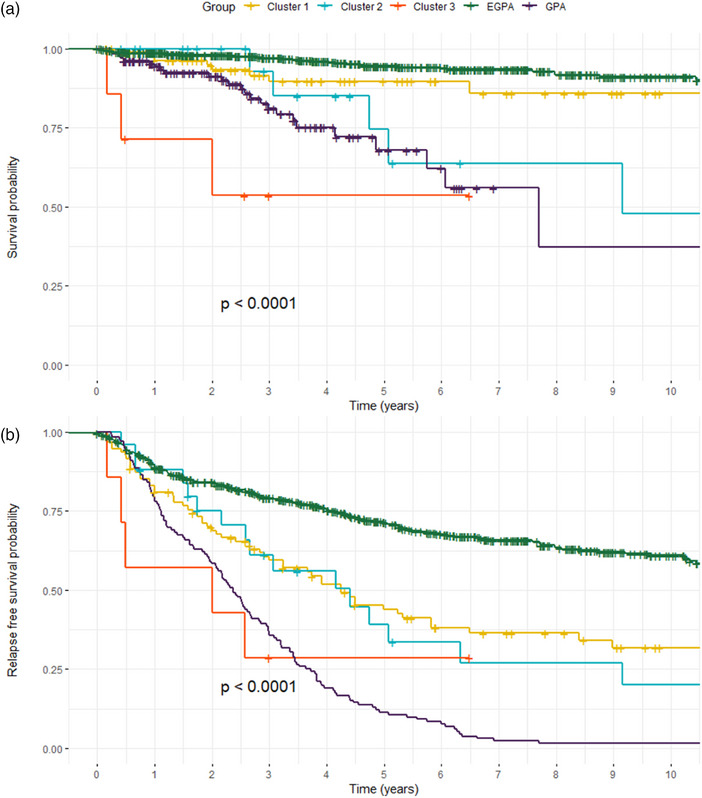
**Overall survival (a) and relapse‐free survival (b) curves in the clusters of** eosinophilic granulomatosis with polyangiitis **(EGPA)/**granulomatosis with polyangiitis **(GPA) overlapping forms patients, and EGPA and GPA control cohorts patients**. Kaplan–Meier survival curves comparing overlap clusters (Clusters 1–3) with typical EGPA and GPA cohorts. (Top) Overall survival probability over a 10‐year follow‐up. (Bottom) Relapse‐free survival probability over a 10‐year follow‐up. Both analyses demonstrate significant differences across groups (log‐rank test, p < 0.0001).

The Kaplan–Meier analysis showed the best OS for Cluster 1 and typical EGPA cohort, whereas Cluster 3 had the poorest survival. RFS was highest in the typical EGPA group and lowest in Cluster 3 and typical GPA cohort (Table S8, Fig. 4).

## Discussion

This study represents the first attempt to comprehensively explore overlapping forms of EGPA and GPA through an unsupervised hierarchical cluster analysis, complemented by comparisons with EGPA and GPA control cohorts. By identifying three distinct clusters within overlapping forms, we provide novel insights into the heterogeneity of these conditions and their clinical implications.

Cluster 1, characterized by a hybrid phenotype of EGPA and GPA, had high rates of asthma (65.7%), PR3‐ANCA positivity (54.5%), and renal involvement (35.4%). This cluster exhibited a higher relapse rate than typical EGPA (RFS at 5 years 49.5% vs. 23.9%), although OS rates were similar (92.6% vs. 96.4% at 5 years). These findings are consistent with previous reports of PR3‐ANCA‐positive EGPA, which highlight a higher risk of renal involvement and relapse [4, 6, 38, 39]. The therapeutic underutilization of rituximab in Cluster 1 (18.2%) may reflect diagnostic uncertainty in overlapping forms, where EGPA and GPA features coexist. PR3‐ANCA positivity, pulmonary nodules or cavitations, and renal involvement support treating these forms as PR3‐AAV, with rituximab as a potentially optimal induction and maintenance therapy [23]. Conversely, anti‐IL‐5/IL‐5R therapies might be considered in patients with predominant asthma or ENT manifestations and eosinophilia, particularly to reduce glucocorticoid dependence [23, 25, 26, 27].

Clusters 2 and 3 were more closely related to GPA phenotypes. Cluster 2, characterized by PR3‐ANCA positivity (78.6%) and frequent renal involvement (75%), represented a less severe yet highly inflammatory form of GPA. The higher prevalence of multiple mononeuropathy compared with typical GPA (42.9% vs. 16%) and underrepresentation of EGPA features such as asthma and urticaria suggest similarities to GPA with eosinophilia, as previously described [15]. The lower proportion of asthma and higher prevalence of nasal crusts and PR3‐ANCA suggest that Cluster 2 patients are more likely to have GPA with asthma rather than EGPA. In our cohort, cyclophosphamide was the primary induction therapy in this cluster, potentially influenced by diagnostic challenges posed by eosinophilia, which may have biased the diagnosis towards EGPA, where the role of rituximab in induction therapy remains less clearly defined [40, 41].

Cluster 3, the smallest and most severe group, was defined by constant renal involvement and diffuse alveolar hemorrhage in more than half of patients (57.1%), and by the poorest outcomes. PR3‐ANCA was present in 71.4% of patients, and this cluster fully overlapped with typical GPA cohort. These patients require management similar to severe GPA cases, underscoring the importance of early and aggressive immunosuppressive therapy, including rituximab and cyclophosphamide.

The a posteriori cluster analysis further validated the consistency of our inclusion criteria, particularly for Cluster 1 and the first definition. These findings underscore potential gaps in the ACR/EULAR 2022 criteria when applied to atypical AAV presentations.

Our findings align with and extend the FAIRVASC consortium's data‐driven subclassification of AAV [42]. FAIRVAS identified five clusters in GPA/MPA patients, improving prognostic prediction compared with diagnosis or ANCA specificity, but excluded EGPA. In our study, Clusters 2 and 3 resemble the PR3‐ANCA kidney involvement cluster, with widespread extrarenal disease and severe kidney cluster, whereas Cluster 1 shows hybrid EGPA/GPA features with no direct counterpart in FAIRVASC. These results emphasize that AAV heterogeneity extends beyond traditional GPA/MPA or PR3/MPO categories and support subclassification approaches that incorporate EGPA and overlap phenotypes. Based on the underlying pathophysiology mechanisms, three main hypotheses can be formulated to explain overlapping forms. First, some patients may represent true EGPA cases with unusually pronounced vasculitic and granulomatous features, thereby mimicking GPA, as previously suggested for PR3‐positive EGPA by Papo et al. [4]. Second, the coexistence of two distinct AAV entities—EGPA and GPA—theoretically occurs, although this appears improbable given their distinct immunopathogenic pathways and genetic backgrounds. Third, overlapping clinical phenotypes may reflect a shared genetic predisposition, possibly involving rare combinations of multiple susceptibility variants. To improve the identification of such cases in clinical practice, additional Th2‐related biomarkers, such as serum IgG4 and IgE levels, may complement eosinophil counts and help discriminate between EGPA and GPA.

This study has several strengths, including its innovative use of hierarchical clustering to explore overlapping AAV phenotypes and the inclusion of validated EGPA and GPA cohorts for robust comparisons. The addition of validated control cohorts of typical EGPA and GPA [4, 43] allowed for a direct comparison of our population and improved identification of patient profiles, supporting our hypothesis of EGPA/GPA overlap, particularly for Cluster 1. The close concordance between the a posteriori clustering and the clinical diagnoses of EGPA and GPA supports the robustness of this approach and highlights the potential value of cluster analysis for future studies in AAV.

However, the retrospective design introduces inherent limitations, such as selection bias and incomplete data. To reduce the risk of selection bias, we applied strict and pragmatic inclusion definitions based on the latest ACR/EULAR 2022 classification for EGPA and GPA [23, 29, 30] and the EMA algorithm [31]. In addition, the impact of the initial diagnosis on global and therapeutic management could not be evaluated due to missing data, limiting the interpretation of the clustering results. Similarly, ANCA titers were not systematically collected in our cohort, and we were therefore unable to assess whether titer changes were associated with disease relapses.

Although some variables included in the multiple correspondence analysis (e.g., purpura and urticaria) are subcategories of broader clinical domains (e.g., cutaneous involvement), we retained them to preserve clinical specificity. We acknowledge that this may introduce conditional dependencies, a known limitation of multiple correspondence analysis analyses, although the FactoMineR package is relatively robust to such correlations.

The small number of patients in Cluster 3 (*n* = 7) further limits the statistical robustness of inter‐group comparisons. Consequently, the higher mortality and RFS trends observed in this cluster should be interpreted with caution. This limitation also affects the reliability of conclusions regarding therapeutic response in this subgroup.

In conclusion, our findings support the existence of overlapping forms between EGPA and GPA, as well as an eosinophilic phenotype of GPA. These distinctions have important clinical implications, particularly for optimizing treatment strategies tailored to individual patient profiles. Future studies with larger cohorts and prospective designs are essential to further define these overlapping forms, assess their long‐term outcomes, and refine diagnostic and therapeutic approaches in AAV.

## Author contributions

*Conceptualization*: Federica Pallotti, Camille Mettler, Benjamin Terrier. Data curation: Federica Pallotti, Camille Mettler, Benjamin Terrier. *Formal analysis/Statistical analysis*: Camille Mettler. *Investigation*: All authors. *Methodology*: Federica Pallotti, Camille Mettler, Benjamin Terrier. *Writing—original draft*: Federica Pallotti, Camille Mettler, Benjamin Terrier. *Writing—review and editing*: All authors. *Supervision*: Benjamin Terrier.

## Funding information

Any author declared a specific grant for this research from any funding agency in the public, commercial, or not‐for‐profit sectors.

## Ethics Statement

This study was approved by the local ethics committee (Comité Local d’éthique pour les publications de l'hôpital Cochin [CLEP]).

## Conflict of interest statement

All the authors declare no conflicts of interest for this study and have nothing to disclose.

## Supporting information




**Table S1**: Inclusion criteria leading to four overlapping form definitions according to ACR/EULAR 2022 classification criteria fulfilling PR3‐ANCA presence, lung nodules, and eosinophilia >1000/mm^3^.
**Table S2**: Characteristics of the patients with EGPA/GPA overlapping forms included in the study, according to the four inclusion definitions.
**Table S3**: Therapeutic management and outcome of the study population.
**Table S4**: Proportion of patients from initial definition of inclusion criteria to cluster groups.
**Table S5**: Histological features according to cluster groups.
**Table S6**: Therapeutic management according to cluster groups after 2010.
**Table S7**: Clinical and biological features during relapse according to cluster groups.
**Table S8**: Characteristics of EGPA‐GPA overlapping forms compared to typical EGPA and GPA cohorts.
**Fig. S1**: Flow chart of EGPA and GPA control cohorts’ selection.
**Fig. S2**: Relapse‐free survival (A) and overall survival (B) of the study population.
**Fig. S3**: Kaplan–Meier curves of relapse‐free survival according to cluster group (A) and Fine‐Gray model for relapse‐free survival according to cluster group (B).
**Fig. S4**: Mean vectors of each cluster across the dimensions of the principal component analysis.

## Data Availability

The data that support the findings of this study are available from the corresponding author upon reasonable request.
