# Supplementary material for: Overlapping forms of granulomatosis with polyangiitis and eosinophilic granulomatosis with polyangiitis: Insights from a European multicenter study
Source: J Intern Med. 2025 Dec 9;299(3):349–64. doi: 10.1111/joim.70056 (PMC12869015; doi:10.1111/joim.70056)
Supplement: Supplementary file 1 — Table S1: Inclusion criteria leading to four overlapping form definitions according to ACR/EULAR 2022 classification criteria fulfilling PR3‐ANCA presence, lung nodules, and eosinophilia >1000/mm3. Table S2: Characteristics of the patients with EGPA/GPA overlapping forms included in the study, according to the four inclusion definitions. Table S3: Therapeutic management and outcome of the study population. Table S4: Proportion of patients from initial definition of inclusion criteria to cluster groups. Table S5: Histological features according to cluster groups. Table S6: Therapeutic management according to cluster groups after 2010. Table S7: Clinical and biological features during relapse according to cluster groups. Table S8: Characteristics of EGPA‐GPA overlapping forms compared to typical EGPA and GPA cohorts. Fig. S1: Flow chart of EGPA and GPA control cohorts’ selection. Fig. S2: Relapse‐free survival (A) and overall survival (B) of the study population. Fig. S3: Kaplan–Meier curves of relapse‐free survival according to cluster group (A) and Fine‐Gray model for relapse‐free survival according to cluster group (B). Fig. S4: Mean vectors of each cluster across the dimensions of the principal component analysis. [file JOIM-299-349-s001.docx]

**Overlapping forms of EGPA and GPA**

Federica Pallotti, Camille Mettler, Matthias Papo, Michele Iudici, Roberto Padoan, Boris Sorin, et al., on behalf of the French Vasculitis Study Group (FVSG).

**Supplementary materials**

**Tables 1 to 8**

**Figures 1 to 4**

Table of Contents

[Supplementary Table 1. Inclusion criteria leading to four overlapping forms definitions according to ACR/EULAR 2022 classification criteria fulfilling, PR3-ANCA presence, lung nodules, and eosinophilia > 1000/mm^3^. 3](#_Toc211185985)

[Supplementary Table 2. Characteristics of the patients with EGPA/GPA overlapping forms included in the study, according to the four inclusion definitions. 4](#_Toc211185986)

[Supplementary Table 3. Therapeutic management and outcome of the study population. 6](#_Toc211185987)

[Supplementary Table 4. Proportion of patients from initial definition of inclusion criteria to cluster groups. 7](#_Toc211185988)

[Supplementary Table 5. Histological features according to cluster groups. 8](#_Toc211185989)

[Supplementary Table 6. Therapeutic management according to cluster groups after 2010. 9](#_Toc211185990)

[Supplementary Table 7. Clinical and biological features during relapse according to cluster groups. 10](#_Toc211185991)

[Supplementary Table 8. Characteristics of EGPA-GPA overlapping forms compared to typical EGPA and GPA cohorts. 11](#_Toc211185992)

[Supplementary Figure 1. Flow chart of EGPA and GPA control cohorts’ selection. 12](#_Toc211185993)

[Supplementary Figure 2. Relapse-free survival (A) and overall survival (B) of the study population. 13](#_Toc211185994)

[Supplementary Figure 3. Kaplan-Meier curves of relapse-free survival according to cluster group (A) and Fine Gray model for relapse-free survival according to cluster group (B). 14](#_Toc211185995)

[Supplementary Figure 4. Mean vectors of each cluster across the dimensions of the principal component analysis. 15](#_Toc211185996)

# Supplementary Table 1. Inclusion criteria leading to four overlapping forms definitions according to ACR/EULAR 2022 classification criteria fulfilling, PR3-ANCA presence, lung nodules, and eosinophilia > 1000/mm^3^.

| **Inclusion criteria** | **ACR/EULAR classification criteria for EGPA** $\boldsymbol{\geq}$ **6** | **ACR/EULAR classification criteria for GPA** $\boldsymbol{\geq}$ **5** | **PR3-ANCA presence** | **GPA surrogate markers** | **Eosinophilia**  $\boldsymbol{\geq}$**1000/mm3** |
| --- | --- | --- | --- | --- | --- |
| **Definition 1** | + | + | +/- | +/- | +/- |
| **Definition 2** | + | - | + | +/- | +/- |
| **Definition 3** | - | + | +/- | +/- | + |
| **Definition 4** | - | - | + | +/- | + |
| *Footnote*: Inclusion criteria were based on the 2022 ACR/EULAR classification criteria for Eosinophilic Granulomatosis with Polyangiitis (EGPA) and Granulomatosis with Polyangiitis (GPA), as well as the presence of PR3-ANCA, lung nodules, and/or eosinophilia >1000/mm³.  GPA surrogate markers, as defined by the EMA algorithm, included: pulmonary nodules or cavitations persisting for >1 month; bronchial stenosis; bloody nasal discharge and crusting for >1 month; nasal ulceration, otitis media, or mastoiditis for >3 months; retro-orbital mass or inflammation; subglottic stenosis; saddle nose deformity; or destructive sinonasal disease.  Definition 1: Patients fulfilling both 2022 ACR/EULAR classification criteria for GPA and EGPA, with or without PR3-ANCA, lung nodules, and/or eosinophilia > 1000/mm3.  Definition 2: Patients meeting the 2022 ACR/EULAR classification criteria for EGPA and with PR3-ANCA and/or surrogate GPA markers.  Definition 3: Patients meeting the 2022 ACR/EULAR criteria for GPA with eosinophils >1000/mm³.  Definition 4: Patients diagnosed with AAV without fulfilling the 2022 ACR/EULAR criteria for EGPA or GPA but exhibited eosinophils >1000/mm³ and PR3-ANCA and/or surrogate GPA markers.  ACR: American College of Rheumatology, ANCA: antineutrophil cytoplasm antibodies, EGPA: eosinophilic granulomatosis with polyangiitis, EULAR: European League Against Rheumatism, GPA: granulomatosis with polyangiitis, PR3-ANCA : anti-proteinase-3 antibodies. | | | | | |

# Supplementary Table 2. Characteristics of the patients with EGPA/GPA overlapping forms included in the study, according to the four inclusion definitions.

|  | Overall  n=135 | Definition 1  n=26 | Definition 2  n=47 | Definition 3  n=45 | Definition 4  n=17 |
| --- | --- | --- | --- | --- | --- |
| Female | 51 (37.8%) | 11 (42.3%) | 22 (46.8%) | 14 (31.1%) | 4 (23.5%) |
| Age at diagnosis (years) | 52.5 [41.2-64] | 49 [43-63.8] | 51.5 [40.2-64] | 51.5 [37.2-58.2] | 65 [51.8-71.5] |
| *ANCA status* |  |  |  |  |  |
| Positive ANCA | 108 (80%) | 23 (88.5%) | 24 (51.1%) | 44 (97.8%) | 17 (100%) |
| PR3-ANCA | 82 (60.7%) | 19 (73.1%) | 11 (23.4%) | 37 (82.2%) | 15 (88.2%) |
| MPO-ANCA | 22 (16.3%) | 2 (7.7%) | 12 (25.5%) | 7 (15.6%) | 1 (5.9%) |
| *Clinical manifestations* |  |  |  |  |  |
| Constitutional symptoms | 95 (70.4%) | 18 (69.2%) | 29 (61.7%) | 35 (77.8%) | 13 (76.5%) |
| Arthralgia | 85 (63%) | 17 (65.4%) | 21 (44.7%) | 35 (77.8%) | 12 (70.6%) |
| Myalgia | 55 (40.7%) | 8 (30.8%) | 16 (34%) | 24 (53.3%) | 7 (41.2%) |
| Lung involvement | 122(90.4%) | 24 (92.3%) | 46 (97.9%) | 36 (80%) | 16 (94.1%) |
| Asthma | 76 (56.3%) | 21 (80.8%) | 44 (93.6%) | 8 (17.8%) | 3 (17.6%) |
| Nodules | 76 (56.3%) | 16 (61.5%) | 35 (74.5%) | 18 (40%) | 7 (41.2%) |
| Consolidation | 57 (42.2%) | 11 (42.3%) | 21 (44.7%) | 20 (44.4%) | 5 (29.4%) |
| Cavitation | 10 (7.4%) | 2 (7.7%) | 2 (4.3%) | 5 (11.1%) | 1 (5.9%) |
| DAH | 27 (20%) | 5 (19.2%) | 4 (8.5%) | 14 (31.1%) | 4 (23.5%) |
| Pleuritis | 11 (8.1%) | 0 (0%) | 7 (14.9%) | 2 (4.4%) | 2 (11.8%) |
| Subglottic stenosis | 5 (3.7%) | 3 (11.5%) | 0 (0%) | 2 (4.4%) | 0 (0%) |
| Bronchial stenosis | 4 (3%) | 2 (7.7%) | 2 (4.3%) | 0 (0%) | 0 (0%) |
| ENT involvement | 114(84.4%) | 26 (100%) | 37 (78.7%) | 44 (97.8%) | 7 (41.2%) |
| Chronic sinusitis | 83 (61.5%) | 20 (76.9%) | 29 (61.7%) | 32 (71.1%) | 2 (11.8%) |
| Nasal crusts | 71 (52.5%) | 16 (61.5%) | 12 (25.5%) | 39 (86.7%) | 4 (23.5%) |
| Nasal polyps | 33 (24.4%) | 14 (53.8%) | 16 (34%) | 3 (6.7%) | 0 (0%) |
| Otologic | 22 (16.3%) | 4 (15.4%) | 6 (12.8%) | 11 (24.4%) | 1 (5.9%) |
| Chondritis | 8 (5.9%) | 5 (19.2%) | 0 (0%) | 3 (6.7%) | 0 (0%) |
| Cutaneous involvement | 70 (51.9%) | 17 (65.4%) | 24 (51.1%) | 23 (51.1%) | 6 (35.3%) |
| Purpura | 38 (28.1%) | 11 (42.3%) | 10 (21.3%) | 12 (26.7%) | 5 (29.4%) |
| Urticaria | 15 (11.1%) | 3 (11.5%) | 8 (17%) | 4 (8.9%) | 0 (0%) |
| Ulcer | 9 (6.7%) | 2 (7.7%) | 2 (4.3%) | 4 (8.9%) | 1 (5.9%) |
| Nodules | 9 (6.7%) | 2 (7.7%) | 4 (8.5%) | 3 (6.7%) | 0 (0%) |
| Livedo | 7 (5.2%) | 2 (7.7%) | 2 (4.3%) | 3 (6.7%) | 0 (0%) |
| Glomerulonephritis | 64 (47.4%) | 7 (26.9%) | 12 (25.5%) | 35 (77.8%) | 10 (58.8%) |
| Serum creatinine >150 μmol/L | 42 (31.1%) | 5 (19.2%) | 7 (14.9%) | 24 (53.3%) | 6 (35.3%) |
| Proteinuria | 55 (40.7%) | 7 (26.9%) | 9 (19.1%) | 34 (75.6%) | 5 (29.4%) |
| Hematuria | 52 (38.5%) | 6 (23.1%) | 6 (12.8%) | 34 (75.6%) | 6 (35.3%) |
| Peripheral neuropathy | 54 (40%) | 10 (38.5%) | 24 (51.1%) | 17 (37.8%) | 3 (17.6%) |
| Multiple mononeuropathy | 39 (28.9%) | 7 (26.9%) | 16 (34%) | 14 (31.1%) | 2 (11.8%) |
| Polyneuropathy | 16 (11.9%) | 4 (15.4%) | 6 (12.8%) | 4 (8.9%) | 2 (11.8%) |
| CNS involvement | 11 (8.1%) | 2 (7.7%) | 4 (8.5%) | 4 (8.9%) | 1 (5.9%) |
| Strokes | 5 (3.7%) | 1 (3.8%) | 3 (6.4%) | 1 (2.2%) | 0 (0%) |
| Cerebral vasculitis | 3 (2.2%) | 0 (0%) | 3 (6.4%) | 0 (0%) | 0 (0%) |
| Pachymeningitis | 3 (2.2%) | 1 (3.8%) | 0 (0%) | 1 (2.2%) | 1 (5.9%) |
| Cardiovascular involvement | 31 (23%) | 3 (11.5%) | 18 (38.3%) | 7 (15.6%) | 3 (17.6%) |
| Myocarditis | 13 (9.6%) | 2 (7.7%) | 8 (17%) | 3 (6.7%) | 0 (0%) |
| Pericarditis | 12 (8.9%) | 1 (3.8%) | 8 (17%) | 2 (4.4%) | 1 (5.9%) |
| Ocular involvement | 24 (17.8%) | 6 (23.1%) | 5 (10.6%) | 8 (17.8%) | 5 (29.4%) |
| Scleritis/episcleritis | 15 (11.1%) | 5 (19.2%) | 1 (2.1%) | 5 (11.1%) | 4 (23.5%) |
| Orbital mass | 1 (0.7%) | 1 (3.8%) | 0 (0%) | 0 (0%) | 0 (0%) |
| Gastrointestinal involvement | 23 (17%) | 4 (15.4%) | 9 (19.1%) | 6 (13.3%) | 4 (23.5%) |
| FFS score $\boldsymbol{\geq}$1 | 30 (22.2%) | 4 (15.4%) | 10 (21.3%) | 6 (13.3%) | 10 (58.8%) |
| Biological features |  |  |  |  |  |
| CRP (mg/L) | 61 [29-113] | 51 [26-107] | 44.5 [15-68] | 79 [46-189] | 96 [46-139] |
| Serum creatinine (µmol/L) | 88 [72-115] | 88 [72-112] | 83 [60-97] | 99 [79-135] | 104 [80-168] |
| Eosinophil count (cells/mm^3^) | 2,400  [1,400-6,000] | 1,900  [1,500-4,400] | 5,200  [2,300-8,700] | 1,900  [1,300-3,200] | 1,400  [1,300-1,700] |
| 2022 ACR/EULAR  classification criteria |  |  |  |  |  |
| EGPA criteria ≥6, n | 72 (53.3%) | 26 (100%) | 47 (100%) | 0 (0%) | 0 (0%) |
| GPA criteria ≥5, n | 66 (48.9%) | 26 (100%) | 0 (0%) | 40 (88.9%)***** | 0 (0%) |
| *Footnote*: Data are presented as median [IQR] or number (proportion).  *Abbreviation*: ANCA: antineutrophil cytoplasm antibodies, CNS: central nervous system, CRP: C-reactive protein, DAH: diffuse alveolar hemorrhage; ENT: ear-nose-throat, EGPA: eosinophilic granulomatosis with polyangiitis, FFS: Five Factor Score, GPA: granulomatosis with polyangiitis. MPO-ANCA: anti-myeloperoxidase antibodies; PR3-ANCA: anti-proteinase-3 antibodies.  ** Of the 45 patients of the definition 3, 5 patients did not meet the 2022 ACR/EULAR criteria for GPA but GPA diagnosis was retained according to the EMEA diagnostic algorithm.* | | | | | |

# Supplementary Table 3. Therapeutic management and outcome of the study population.

|  | Overall  (n=135) |
| --- | --- |
| *Induction therapy* |  |
| Glucocorticoids | 134 (99.3%) |
| Pulses of methylprednisolone | 65 (48.1%) |
| Oral prednisone, median dose | 60 [50-60] |
| Main immunosuppressive agents | 100 (74.1%) |
| Cyclophosphamide | 57 (42.2%) |
| Rituximab | 23 (17%) |
| Methotrexate | 8 (5.9%) |
| Azathioprine | 7 (5.2%) |
| Mepolizumab | 3 (2.2%) |
| Plasma exchanges | 11 (8.1%) |
| *Maintenance therapy* |  |
| Oral prednisone | 77 (57%) |
| Median prednisone-equivalent oral dose (mg/day) | 5 [0-5] |
| Azathioprine | 40 (29.6%) |
| Rituximab | 20 (14.8%) |
| Methotrexate | 19 (14.1%) |
| Mepolizumab | 6 (4.4%) |
| Mycophenolate mofetil | 2 (1.5%) |
| *Main additional treatments required during follow-up* | |
| Rituximab | 27 (20%) |
| Mycophenolate mofetil | 15 (11.1%) |
| Azathioprine | 11 (8.1%) |
| Mepolizumab | 11 (8.1%) |
| Intravenous cyclophosphamide | 9 (6.7%) |
| Oral cyclophosphamide | 9 (6.7%) |
| Methotrexate | 8 (5.9%) |
| Plasma exchange | 5 (3.7%) |
| Benralizumab | 3 (2.2%) |
| Avacopan | 1 (0.7%) |
| Dupilumab | 1 (0.7%) |
| *Outcomes* |  |
| Mean follow-up duration (months) | 77.9 [40.5-121.2] |
| Remission | 126 (93.3%) |
| Relapse | 73 (54.1%) |
| Major relapse | 48 (35.6%) |
| Minor relapse | 12 (8.9%) |
| Isolated asthma-ENT relapses | 13 (9.6%) |
| GCs dependency ≥5 mg/day | 65 (48.1%) |
| Relapse-free survival |  |
| 1-year | 105 (82%) |
| 3-year | 77 (60.2%) |
| 5-year | 61 (47.7%) |
| Overall survival |  |
| 1-year | 124 (97.6%) |
| 3-year | 117 (92.1%) |
| 5-year | 114 (89.8%) |
| Deaths | 18 (13.3%) |
| *Footnote*: Data are presented as median [IQR] or number (proportion).  *Abbreviations*: ENT: ear-nose-throat, GCs: glucocorticoids, IV: intravenous. | |

# Supplementary Table 4. Proportion of patients from initial definition of inclusion criteria to cluster groups.

|  | **Cluster 1**  **(n=99)** | **Cluster 2**  **(n=28)** | **Cluster 3**  **(n=7)** |
| --- | --- | --- | --- |
| **Initial definition of inclusion criteria** |  |  |  |
| Definition 1 (n=26) | 21 (81%) | 4 (15.4%) | 1 (3.8%) |
| Definition 2 (n=47) | 43 (91.5%) | 3 (6.4%) | 1 (2.1%) |
| Definition 3 (n=45) | 25 (55.6%) | 16 (35.6%) | 4 (8.9%) |
| Definition 4 (n=17) | 10 (58.8%) | 5 (29.4%) | 1 (5.9%) |
| *Footnote*: Definition 1= Both GPA and EGPA criteria; Definition 2= EGPA criteria and PR3-ANCA +/- lung nodules; Definition 3 = GPA criteria and eosinophilia > 1000/mm^3^; Definition 4 = AAV diagnosis with PR3-ANCA and eosinophilia > 1000/mm^3^ | | | |

# Supplementary Table 5. Histological features according to cluster groups.

|  | **Cluster 1 (n=99)** | **Cluster 2 (n=28)** | **Cluster 3 (n=7)** | |  |
| --- | --- | --- | --- | --- | --- |
| **Biopsy** | 57 (57.6%) | 19 (67.9%) | 6 (85.7%) | |  |
| Skin | 27 (27.3%) | 4 (14.3%) | 1 (14.3%) | |  |
| Lung | 13 (13.1%) | 2 (7.1%) | 0 (0%) | |  |
| Kidney | 16 (16.2%) | 9 (32.1%) | 6 (85.7%) | |  |
| Sinus | 13 (13.1%) | 3 (10.7%) | 3 (42.9%) | |  |
| Gut | 3 (3%) | 1 (3.6%) | 0 (0%) | |  |
| Nerve | 10 (10.1%) | 3 (10.7%) | 1 (14.3%) | |  |
| Other | 2 (2%) | 1 (3.6%) | 0 (0%) | |  |
| **Histologic findings** |  |  |  | |  |
| Granuloma | | 11 (19.3%) | 6 (31.6%) | 2 (33.3%) |  |
| Necrosis | | 24 (42.1%) | 10 (52.6%) | 3 (50%) |  |
| Eosinophilic infiltrates | | 19 (33.3%) | 1 (5.3%) | 2 (33.3%) |  |
| Extra-capillary glomerulonephritis | | 13 (81.3%) | 9 (100%) | 6 (100%) |  |
| **Histological conclusion: vasculitis** | 41 (71.9%) | 17 (89.5%) | 6 (100%) | |  |
| **Histological conclusion: GPA** | 16 (28.1%) | 8 (42.1%) | 3 (50%) | |  |
| **Histological conclusion: EGPA** | 16 (28.1%) | 5 (26.3%) | 2 (33.3%) | |  |
| **Histological conclusion: non-specific** | 13 (22.8%) | 1 (5.3%) | 0 (0%) | |  |
| *Footnote*: Data are presented as number (proportion). | | | | | |

# Supplementary Table 6. Therapeutic management according to cluster groups after 2010.

|  | **Cluster 1 (n=59)** | **Cluster 2 (n=17)** | **Cluster 3 (n=1)** |
| --- | --- | --- | --- |
| **Induction treatment** |  |  |  |
| Glucocorticoids | 57 (96.6%) | 16 (94.1%) | 1 (100%) |
| Methylprednisolone pulses (n) | 25 (42.4%) | 13 (76.5%) | 1 (100%) |
| Cyclophosphamide | 13 (22%) | 9 (52.9%) | 1 (100%) |
| Rituximab | 18 (30.5%) | 5 (29.4%) | 0 (0%) |
| Plasma exchange | 1 (1.7%) | 3 (17.6%) | 1 (100%) |
| Azathioprine | 6 (10.2%) | 0 (0%) | 0 (0%) |
| Methotrexate | 6 (10.2%) | 1 (5.9%) | 0 (0%) |
| Mepolizumab | 2 (3.4%) | 1 (5.9%) | 0 (0%) |
| **Maintenance treatment** |  |  |  |
| Rituximab | 12 (20.3%) | 5 (29.4%) | 0 (0%) |
| Azathioprine | 17 (28.8%) | 3 (17.6%) | 0 (0%) |
| Methotrexate | 11 (18.6%) | 1 (5.9%) | 0 (0%) |
| Mepolizumab | 5 (8.5%) | 1 (5.9%) | 0 (0%) |
| Median glucocorticoid oral dose (mg/day) | 5 [0-5] | 0 [0-5] | 5 [5-5] |
| *Footnote*: Data are presented as median [IQR] or number (proportion). | | | |

# Supplementary Table 7. Clinical and biological features during relapse according to cluster groups.

|  | **Cluster 1**  **(n=99)** | **Cluster 2**  **(n=28)** | **Cluster 3**  **(n=7)** |
| --- | --- | --- | --- |
| **Number of relapses** | 57 | 13 | 3 |
| **Pulmonary** | 31 (54.4%) | 8 (61.5%) | 2 (66.7%) |
| Nodules | 3 (5.3%) | 4 (30.8%) | 2 (66.7%) |
| Asthma | 19 (33.3%) | 2 (15.4%) | 0 (0%) |
| Lung consolidation | 3 (5.3%) | 0 (0%) | 0 (0%) |
| Diffuse alveolar hemorrhage | 3 (5.3%) | 0 (0%) | 0 (0%) |
| Others | 13 (22.8%) | 1 (7.7%) | 1 (33.3%) |
| **ENT** | 26 (45.6%) | 6 (46.2%) | 2 (66.7%) |
| Nasal crusts | 12 (21.1%) | 5 (38.5%) | 0 (0%) |
| Sinusitis | 13 (22.8%) | 3 (23.1%) | 0 (0%) |
| Nasal polyposis | 4 (7%) | 0 (0%) | 0 (0%) |
| Otologic | 5 (8.8%) | 1 (7.7%) | 1 (33.3%) |
| Stenosis | 3 (5.3%) | 0 (0%) | 1 (33.3%) |
| ENT-other | 3 (5.3%) | 2 (15.4%) | 0 (0%) |
| **Arthralgia** | 15 (26.3%) | 4 (30.8%) | 2 (66.7%) |
| **Skin** | 11 (19.3%) | 2 (15.4%) | 0 (0%) |
| **Kidney** | 8 (14%) | 3 (23.1%) | 1 (33.3%) |
| **Myopericarditis** | 3 (5.3%) | 3 (23.1%) | 0 (0%) |
| **Multiple mononeuropathy** | 9 (15.8%) | 5 (38.5%) | 2 (66.7%) |
| **Central nervous system** | 2 (3.5%) | 2 (10.5%) | 1 (16.7%) |
| **Digestive** | 6 (10.5%) | 0 (0%) | 0 (0%) |
| **Biology** |  |  |  |
| **PR3-ANCA** | 18 (31.6%) | 8 (61.5%) | 1 (33.3%) |
| **MPO-ANCA** | 4 (7%) | 1 (7.7%) | 1 (33.3%) |
| **CRP (mg/L)** | 0 [0-8] | 10 [0-49] | NA |
| **Serum creatinine (µmol/L)** | 66 [0-90] | 77 [0-100] | 140 [103-150] |
| **Eosinophil count (cells/mm^3^)** | 0 [0-1,000] | 0 [0-0] | 0 [0-600] |
| *Footnote*: Data are presented as median [IQR] or number (proportion). | | | |

# Supplementary Table 8. Characteristics of EGPA-GPA overlapping forms compared to typical EGPA and GPA cohorts.

|  | **Cluster 1**  **n=99** | **Cluster 2**  **n=28** | **Cluster 3**  **n=7** | **Typical EGPA**  **n=656** | **Typical GPA**  **n=229** |
| --- | --- | --- | --- | --- | --- |
| **Gender (female)** | 42 (42.4%) | 8 (28.6%) | 1 (14.3%) | 365 (55.6%) | 97 (42.4%) |
| **Age at diagnosis (years)** | 50 [39.5-64] | 55 [48-64.5] | 44 [35-55.5] | 52 [41-62] | 56 [44-64] |
| **Systemic signs** | 59 (59.6%) | 28 (100%) | 7 (100%) | 423 (64.5%) | 160 (69.9%) |
| **Pulmonary** | 94 (94.9%) | 21 (75%) | 6 (85.7%) | 640 (97.6%) | 156 (68.1%) |
| Nodules / exc. | 61 (61.6%) | 12 (42.9%) | 3 (42.9%) | 0 (0%) | 47 (20.5%) |
| Asthma | 65 (65.7%) | 8 (28.6%) | 3 (42.9%) | 618 (94.2%) | 11 (4.8%) |
| DAH | 17 (17.2%) | 5 (17.9%) | 4 (57.1%) | 12 (1.8%) | 42 (18.3%) |
| Condensations | 44 (44.4%) | 8 (28.6%) | 4 (57.1%) | 337 (51.4%) | 141 (61.6%) |
| **ENT** | 83 (83.8%) | 24 (85.7%) | 6 (85.7%) | 562 (85.7%) | 191 (83.4%) |
| Rhinitis-Nasal crusts | 43 (43.4%) | 17 (60.7%) | 2 (28.6%) | 304 (46.3%) | 146 (63.8%) |
| Sinusitis | 64 (64.6%) | 15 (53.6%) | 4 (57.1%) | 526 (80.2%) | 110 (48%) |
| Otological | 15 (15.2%) | 6 (21.4%) | 1 (14.3%) | 38 (5.8%) | 79 (34.5%) |
| **Arthralgia** | 54 (54.5%) | 26 (92.9%) | 4 (57.1%) | 189 (28.8%) | 129 (56.3%) |
| **Skin** | 51 (51.5%) | 13 (46.4%) | 5 (71.4%) | 230 (35.1%) | 65 (28.4%) |
| Urticaria | 15 (15.2%) | 0 (0%) | 0 (0%) | 61 (9.3%) | 1 (0.4%) |
| Purpura | 24 (24.2%) | 11 (39.3%) | 2 (28.6%) | 121 (18.4%) | 47 (20.5%) |
| Livedo | 5 (5.1%) | 2 (7.1%) | 0 (0%) | 22 (3.4%) | 14 (6.1%) |
| Subcutaneous nodules | 7 (7.1%) | 0 (0%) | 2 (28.6%) | 47 (7.2%) | 9 (3.9%) |
| **Kidney** | 35 (35.4%) | 21 (75%) | 7 (100%) | 84 (12.8%) | 155 (67.7%) |
| **Myopericarditis** | 18 (18.2%) | 2 (7.1%) | 2 (28.6%) | 146 (22.3%) | 15 (6.6%) |
| **Multiple mononeuropathy** | 26 (26.3%) | 12 (42.9%) | 1 (14.3%) | 255 (38.9%) | 35 (15.3%) |
| **CNS** | 9 (9.1%) | 1 (3.6%) | 1 (14.3%) | 29 (4.4%) | 26 (11.4%) |
| **Gastrointestinal** | 16 (16.2%) | 4 (14.3%) | 2 (28.6%) | 107 (16.3%) | 17 (7.4%) |
| **PR3-ANCA** | 54 (54.5%) | 22 (78.6%) | 5 (71.4%) | 0 (0%) | 216 (94.3%) |
| **MPO-ANCA** | 16 (16.2%) | 5 (17.9%) | 1 (14.3%) | 182 (27.7%) | 8 (3.5%) |
| **CRP (mg/L)** | 34 [9-64] | 188.5 [149-217] | 56 [11-83] | 42 [13-87] | 80 [23-168] |
| **Creatinine (µmol/L)** | 79 [56-97] | 102 [81-114] | 477 [347-562] | 76 [55-88] | 86 [67-158] |
| **Eosinophil count (cells/mm^3^)** | 2,400 [1,400-7,000] | 1,900 [1,400-4,000] | 2,100 [1,700-5,700] | 2,600 [1,200-7,600] | 200 [100-400] |
| ***Outcomes*** |  |  |  |  |  |
| **Relapses** | 73 (54.5%) | 57 (57.6%) | 13 (46.4%) | 181 (27.6%) | 119 (52%) |
| **Relapse-free survival** |  |  |  |  |  |
| 1 year | 79 (83.2%) | 22 (88%) | 4 (57.1%) | 560 (89.7%) | 105 (79.5%) |
| 3 years | 59 (62.1%) | 16 (64%) | 2 (28.6%) | 503 (80.6%) | 49 (37.1%) |
| 5 years | 47 (49.5%) | 12 (48%) | 2 (28.6%) | 469 (75.2%) | 15 (11.4%) |
| **Global survival** |  |  |  |  |  |
| 1 year *(n)* | 94 (98.9%) | 25 (100%) | 5 (71.4%) | 616 (98.7%) | 126 (95.5%) |
| 3 years *(n)* | 89 (93.7%) | 24 (96%) | 4 (57.1%) | 609 (97.6%) | 115 (87.1%) |
| 5 years *(n)* | 88 (92.6%) | 22 (88%) | 4 (57.1%) | 600 (96.2%) | 110 (83.3%) |
| **Deaths** | 9 (9.1%) | 5 (17.9%) | 3 (42.9%) | 36 (5.5%) | 28 (12.2%) |
| *Footnote*: Data are presented as median [IQR] or number (proportion).  *Abbreviations*: ANCA: antineutrophil cytoplasm antibodies, CNS: central nervous system, CRP: C-reactive protein, ENT: ear-nose-throat, EGPA: eosinophilic granulomatosis with polyangiitis, FFS: Five Factor Score, GPA: granulomatosis with polyangiitis. MPO-ANCA : anti-myeloperoxidase antibodies ; PR3-ANCA : anti-proteinase-3 antibodies. | | | | | |
|  |  |  |  |  |  |
|  |  |  |  |  |  |

# Supplementary Figure 1. Flow chart of EGPA and GPA control cohorts’ selection.


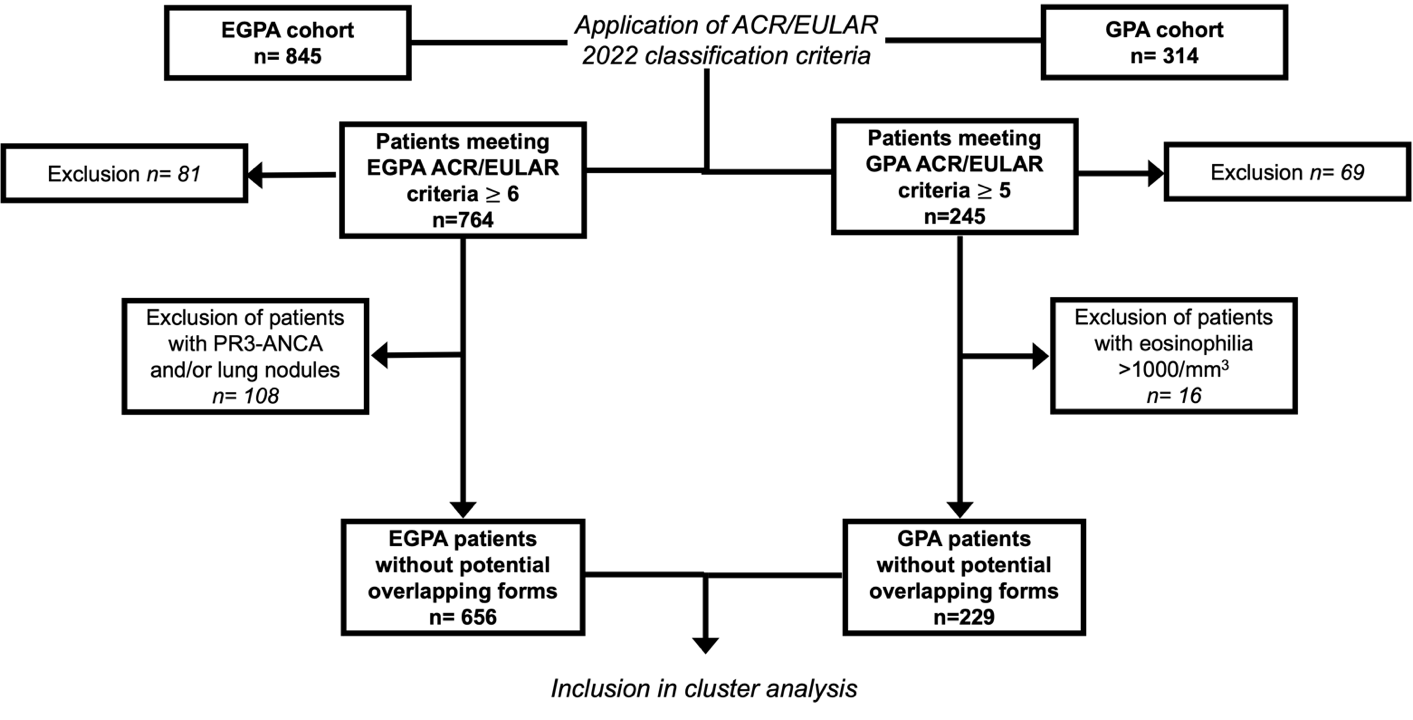


*Abbreviations*: ACR: American College of Rheumatology, EGPA: eosinophilic granulomatosis with polyangiitis, EULAR: European Alliance of Associations for Rheumatology, FVSG: French Vasculitis Study Group, GPA: granulomatosis with polyangiitis.

# Supplementary Figure 2. Relapse-free survival (A) and overall survival (B) of the study population.


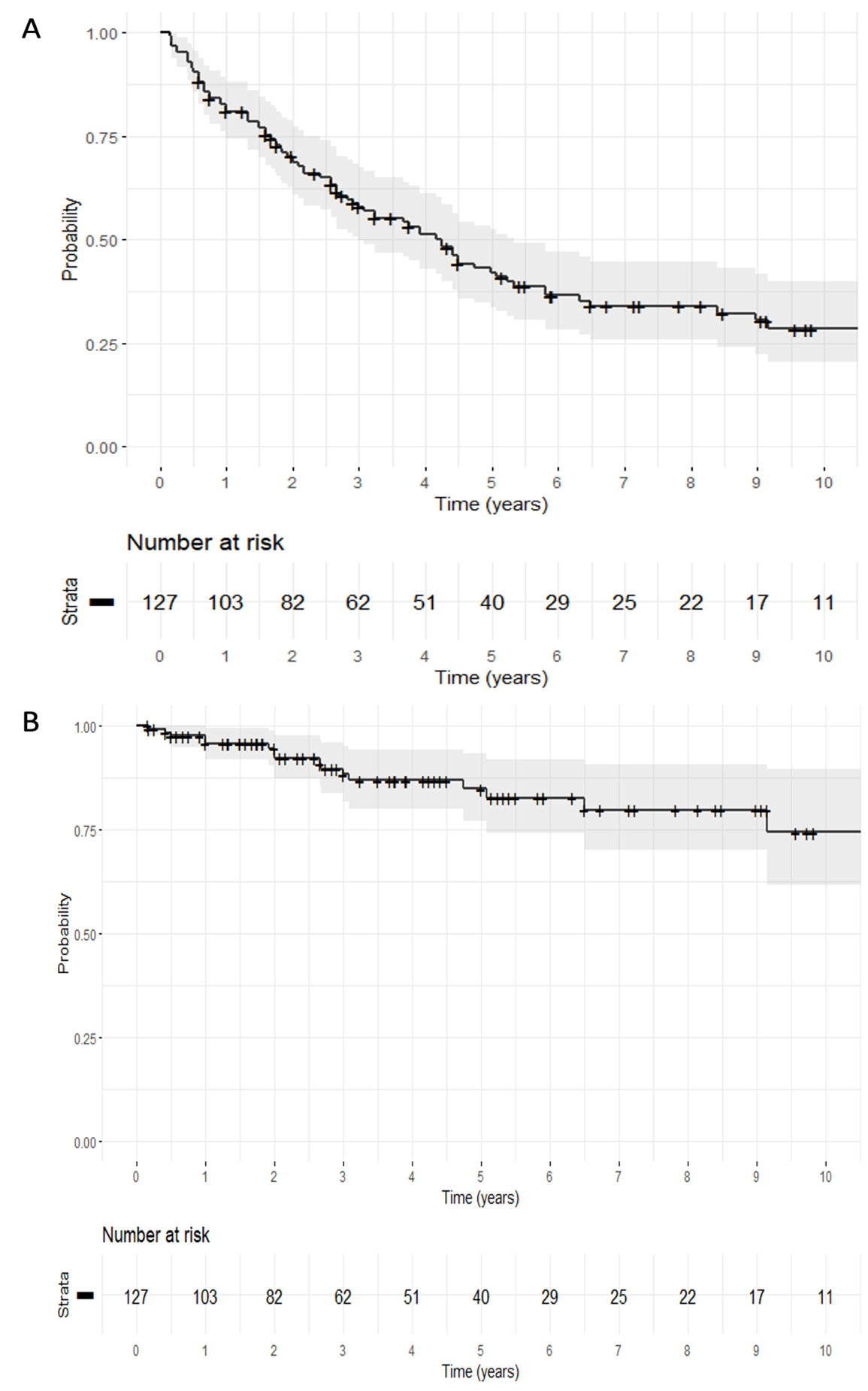


*Legend*: Kaplan-Meier survival curves for the relapse-free survival (panel A) and overall survival (panel B), showing the probability of survival over time. The shaded areas represent the 95% confidence intervals. The number of patients at risk is indicated at the bottom of each panel.

#
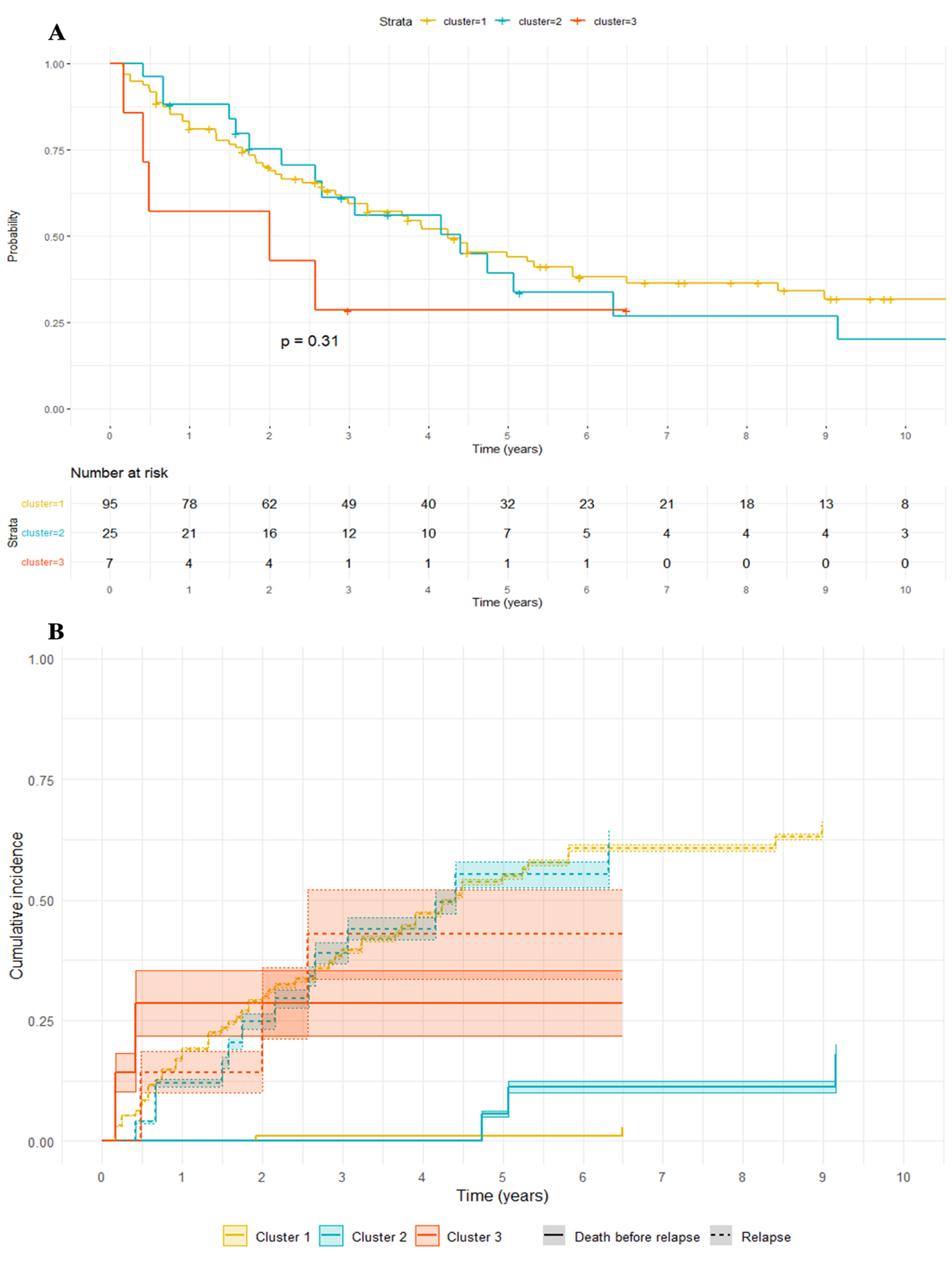
Supplementary Figure 3. Kaplan-Meier curves of relapse-free survival according to cluster grou
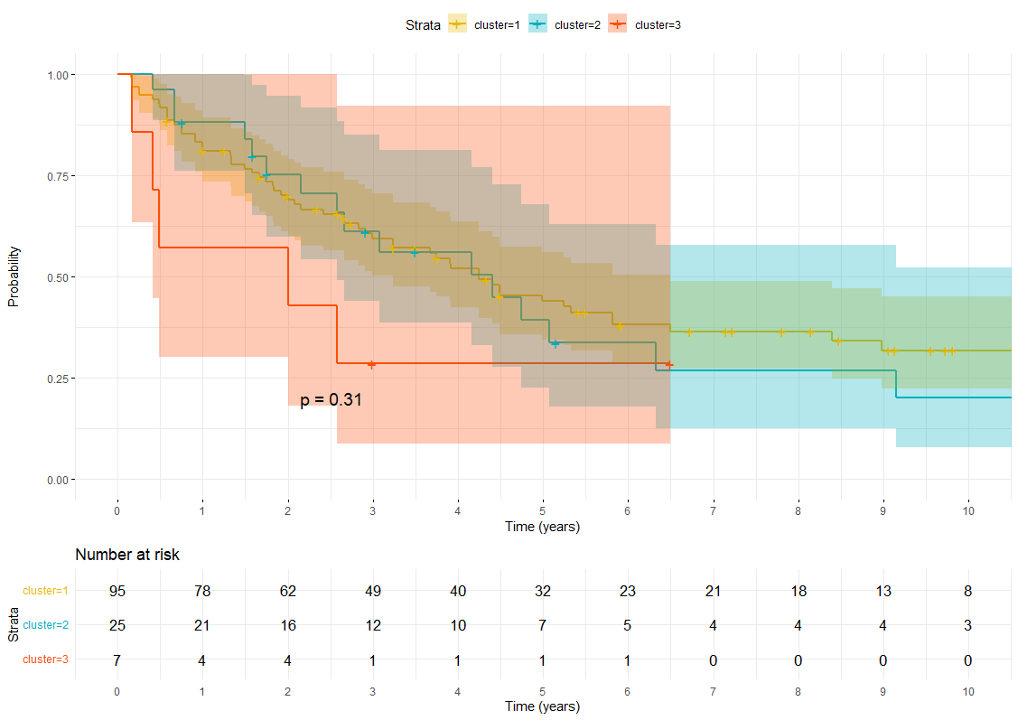
p (A) and
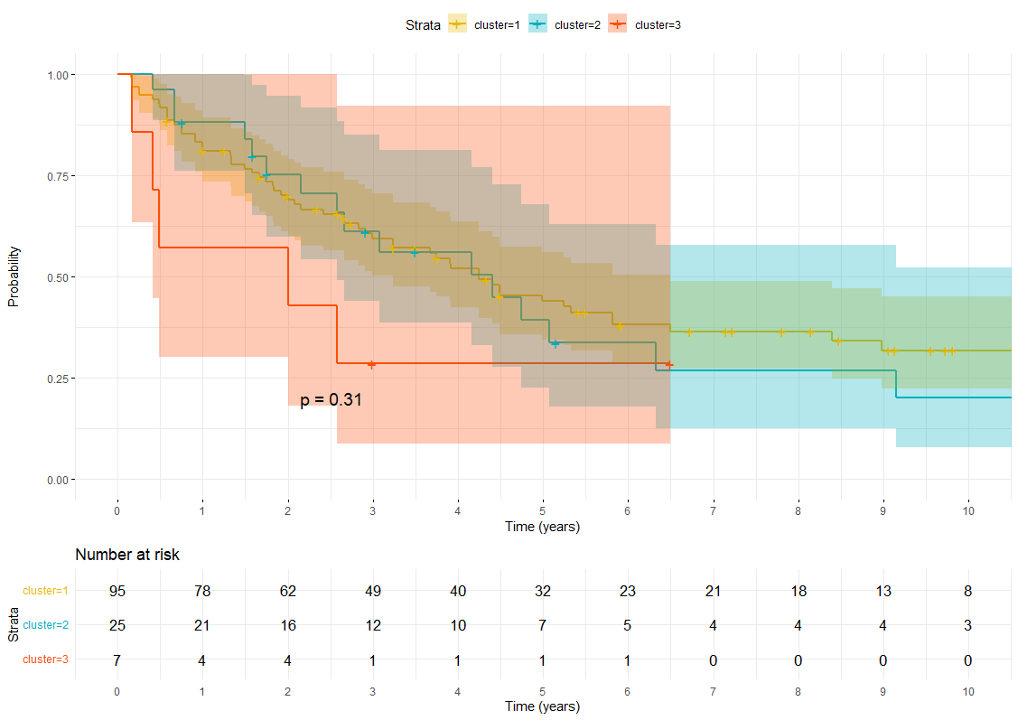
Fine Gray model for relapse-free survival according to cluster group (B).

*Legend*: Kaplan-Meier survival curves (panel A) and Fine-Gray model cumulative incidence curves (panel B) for relapse-free survival stratified by cluster group. In panel A, survival probabilities over time are shown for Clusters 1 (yellow), 2 (blue), and 3 (red), with the number of patients at risk indicated at the bottom. Panel B displays the cumulative incidence of relapse and death before relapse.

# Supplementary Figure 4. Mean vectors of each cluster across the dimensions of the principal component analysis.


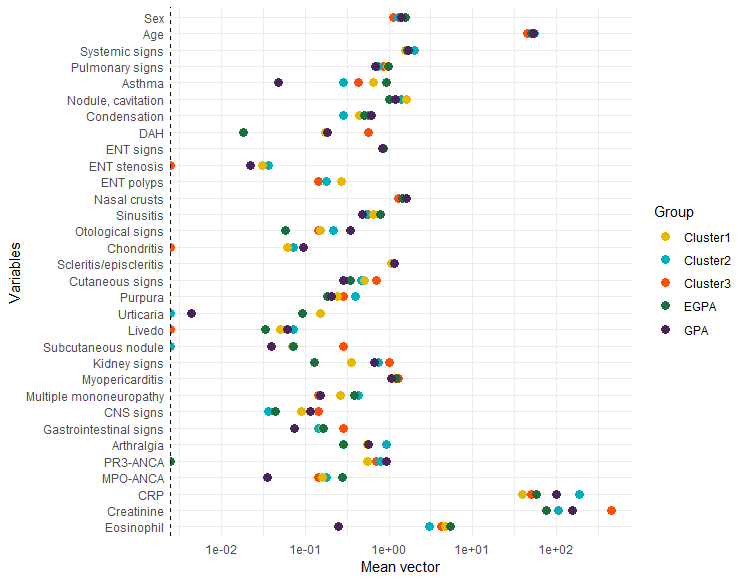


*Legend*: Dot plot depicting the mean vector values of clinical and laboratory variables across different groups: Cluster 1 (yellow), Cluster 2 (cyan), Cluster 3 (red), EGPA (green), and GPA (purple). Each dot represents the mean value of a variable within each group. Variables are listed on the y-axis, and the mean vector is displayed on the x-axis in logarithmic scale. This plot illustrates the differences in variable profiles across the clusters and disease groups, with notable variations in markers such as PR3-ANCA, creatinine, asthma.

*Abbreviations*: CNS, central nervous system; CRP, C-reactive protein; DAH, diffuse alveolar hemorrhage; EGPA, eosinophilic granulomatosis with polyangiitis, ENT, ear-nose-throat; GPA, granulomatosis with polyangiitis; MPO-ANCA, anti-myeloperoxidase antibodies ; PR3-ANCA: anti-proteinase-3 antibodies.
